# Gibbs-Slice Sampling Algorithm for Estimating the Four-Parameter Logistic Model

**DOI:** 10.3389/fpsyg.2020.02121

**Published:** 2020-09-18

**Authors:** Jiwei Zhang, Jing Lu, Hang Du, Zhaoyuan Zhang

**Affiliations:** ^1^Key Lab of Statistical Modeling and Data Analysis of Yunnan Province, School of Mathematics and Statistics, Yunnan University, Kunming, China; ^2^Key Laboratory of Applied Statistics of MOE, School of Mathematics and Statistics, Northeast Normal University, Changchun, China; ^3^School Affiliated to Longhua Institute of Educational Science, Shenzhen, China; ^4^School of Mathematics and Statistics, Yili Normal University, Yili, China

**Keywords:** Bayesian inference, four-parameter logistic model, item response theory, model assessment, potential scale reduction factor, slice sampling algorithm

## Abstract

The four-parameter logistic (4PL) model has recently attracted much interest in educational testing and psychological measurement. This paper develops a new Gibbs-slice sampling algorithm for estimating the 4PL model parameters in a fully Bayesian framework. Here, the Gibbs algorithm is employed to improve the sampling efficiency by using the conjugate prior distributions in updating asymptote parameters. A slice sampling algorithm is used to update the 2PL model parameters, which overcomes the dependence of the Metropolis–Hastings algorithm on the proposal distribution (tuning parameters). In fact, the Gibbs-slice sampling algorithm not only improves the accuracy of parameter estimation, but also enhances sampling efficiency. Simulation studies are conducted to show the good performance of the proposed Gibbs-slice sampling algorithm and to investigate the impact of different choices of prior distribution on the accuracy of parameter estimation. Based on Markov chain Monte Carlo samples from the posterior distributions, the deviance information criterion and the logarithm of the pseudomarginal likelihood are considered to assess the model fittings. Moreover, a detailed analysis of PISA data is carried out to illustrate the proposed methodology.

## 1. Introduction

Over the past four decades, item response theory (IRT) models have been extensively used in educational testing and psychological measurement (Lord and Novick, 1968; Van der Linden and Hambleton, 1997; Embretson and Reise, 2000; Baker and Kim, 2004). These are latent variable modeling techniques, in which the response probability is used to construct the interaction between an individual's “ability” and item level stimuli (difficulty, guessing, etc.), where the focus is on the pattern of responses rather than on composite or total score variables and linear regression theory. Specifically, IRT attempts to model individual ability using question-level performance instead of aggregating test-level performance, and it focuses more on the information provided by an individual on each question. In social sciences, IRT has been applied to attachment (Fraley et al., 2000), personality (Ferrando, 1994; Steinberg and Thissen, 1995; Gray-Little et al., 1997; Rouse et al., 1999), psychopathology (Reise and Waller, 2003; Loken and Rulison, 2010; Waller and Reise, 2010; Waller and Feuerstahler, 2017), attention deficit hyperactivity disorder (Lanza et al., 2005), and delinquency (Osgood et al., 2002), among others.

To explore these applications, it is necessary to establish how the appropriate IRT models should be built and what valuable educational psychological phenomena can be examined to guide practice. In the field of dichotomous IRT models, the one-parameter logistic (1PL) model and the Rasch model (Rasch, 1960), as well as their extensions, the two-parameter logistic model (2PL) (Birnbaum, 1957) and the three-parameter logistic model (3PL) (Birnbaum, 1968), have attracted increasing attention in recent years because of their attractive mathematical properties. However, compared with the widely used 1PL, 2PL, and 3PL models, the four-parameter logistic (4PL) model has languished in obscurity for nearly 30 years (Barton and Lord, 1981), although its importance has gradually been realized by many researchers over the past decade (Hessen, 2005; Loken and Rulison, 2010; Waller and Reise, 2010; Green, 2011; Liao et al., 2012; Yen et al., 2012; Magis, 2013; Waller and Feuerstahler, 2017). This growing interest can be attributed to the need to deal with a number of problems encountered in educational psychology, which can be explained well and indeed solved using the 4PL model. For example, in computerized adaptive testing (CAT), high-ability examinees might on occasion miss items that they should be able to answer correctly, owing to a number of reasons, including anxiety, carelessness, unfamiliarity with the computer environment, distraction by poor testing conditions, or even misreading of the question (Hockemeyer, 2002; Rulison and Loken, 2009). Chang and Ying (2008) demonstrated that the ability determined using the traditional 2PL model is underestimated when the examinee mistakenly answers several items at the beginning of the CAT. In addition, Rulison and Loken (2009) found that using the 3PL model could severely penalize a high-ability examinee who makes a careless error on an easy item (Barton and Lord, 1981; Rulison and Loken, 2009). In psychopathology studies, researchers found that subjects with severe psychopathological disorders may be reluctant to self-report their true attitudes, behaviors, and experiences, so it is obviously inappropriate to use the traditional 3PL model with lower asymptotic parameter to explain such behaviors (Reise and Waller, 2003; Waller and Reise, 2010). Descriptions of the applications of the 4PL model in other areas can be found in Osgood et al. (2002) and Tavares et al. (2004). In addition to the development of the 4PL model in terms of its applications, its theoretical properties have been investigated in some depth. For example, Ogasawara (2012) discussed the asymptotic distribution of the ability, and Magis (2013) systematically studied the properties of the information function and proposed a method for determining its maximum point.

The main reason why the 4PL model has not been more widely used is that an upper asymptotic parameter is added to the 3PL model, which makes parameter estimation more difficult. However, with the rapid development of computer technology in recent years, the estimation problem for complex models has been solved. At the same time, the development of statistical software makes it easier for psychometricians to study complex models such as the 4PL model. Several researchers have used existing software to estimate the 4PL model. For example, Waller and Feuerstahler (2017) investigated 4PL model item and person parameter estimations using marginal maximum likelihood (MML) with the *mirt* (Chalmers, 2012) package, which uses MML via the expectation-maximization (EM) algorithm to estimate simple item response theory models. This is a different approach to that adopted here, where we use a Gibbs-slice sampling algorithm based on augmented data (auxiliary variables). Our Gibbs-slice sampling algorithm is in a fully Bayesian framework, and the posterior samples are drawn from the full conditional posterior distribution, whereas the MML–EM algorithm used in the *mirt* package is in a frequentist framework. Parameter estimates are obtained by an integral operation in the process of implementing the EM algorithm. Loken and Rulison (2010) used WinBUGS (Spiegelhalter et al., 2003) to estimate the 4PL model parameters in a Bayesian framework. However, convergence of parameter estimation is not completely achieved in the case of some non-informative prior distributions for WinBUGS. The reason for this may be that WinBUGS does not explicitly impose the monotonicity restriction *c* < *d* on the 4PL model, i.e., it does not assume that the lower asymptote parameter *c* is smaller than the upper asymptote parameter *d*. (The introduction of parameters in the 4PL model will be described in section 2, and further discussion of these two parameters can be found in Culpepper, 2016 and Junker and Sijtsma, 2001). Thus, the prior Gibbs samplers do not strictly enforce an identification condition, and this leads to estimator non-convergence. More specifically, the prior distributions of the upper and lower asymptote parameters are given by the following informative priors (Loken and Rulison, 2010, p. 513):

$$c_{j}\sim N\left(0.22,0.05\right),\;d_{j}\sim N\left(0.84,0.05\right).$$

If we choose the non-informative prior distributions

$$c_{j}\sim N\left(0.22,10^{5}\right),\;d_{j}\sim N\left(0.84,10^{5}\right),$$

then, from the value ranges of the upper and lower asymptote parameters, we find that the lower asymptote parameter can be larger than the upper asymptote parameter, *d*_*j*_ < *c*_*j*_, which violates the model identification condition *c*_*j*_ < *d*_*j*_ (this condition will be introduced in detail in section 2). In this case, using WinBUGS to infer the model parameters may lead to biased estimates when the sample size (the number of examinees) is small and the prior distributions then play an important role. To solve the above problems in using WinBUGS, Loken and Rulison (2010) employed strong informative prior distributions to obtain good recovery (Culpepper, 2016, p. 1,143). However, Culpepper (2016, p. 1,161) pointed out that the use of informative prior distribution may lead to serious deviations if it happens to be centered at the wrong values. Therefore, he proposed that recovery should also be dealt with by using some non-informative priors.

In the present study, a novel and highly effective Gibbs-slice sampling algorithm in the Bayesian framework is proposed to estimate the 4PL model. The Gibbs-slice sampling algorithm overcomes the defects of WinBUGS that affect the convergence of parameter estimation based on the monotonicity restriction. Moreover, the algorithm can obtain good recovery results by using various types of prior distribution. In the following sections, we will introduce the theoretical foundation of the slice sampling algorithm in detail, and we will then analyze the advantages of the slice sampling algorithm over two traditional Bayesian algorithms.

The rest of this paper is organized as follows. Section 2 contains a short introduction to the 4PL model, its reparameterized form, and model identification restrictions. In section 3, the theoretical foundation of the slice sampling algorithm is presented and its advantages compared with traditional Bayesian algorithms are analyzed. In section 4, three simulation studies focus respectively on the performance of parameter recovery, an analysis of the flexibility and sensitivity of different prior distributions for the slice sampling algorithm, and an assessment of model fittings using two Bayesian model selection criteria. In section 5, the quality of the Gibbs-slice sampling algorithm is investigated using an empirical example. We conclude the article with a brief discussion in section 6.

## 2. Models and Model Identifications

The 1PL and 2PL models have been widely used to fit binary item response data. Birnbaum (1968) modified the 2PL model to give the now well-known 3PL model, which includes a lower asymptote parameter to represent the contribution of guessing to the probability of correct response. To characterize the failure of high-ability examinees to answer easy items, Barton and Lord (1981) introduced an upper asymptote parameter into the 3PL model, giving the 4PL model:

(1) $$\begin{array}{l} P_{ij}=P\left(y_{ij}=1|a_{j},b_{j},c_{j},d_{j},\theta_{i}\right)\\ \begin{array}{l} \;=c_{j}+\left(d_{j}-c_{j}\right)\frac{\text{exp}\left[1.7a_{j}\left(\theta_{i}-b_{j}\right)\right]}{1+\text{exp}\left[1.7a_{j}\left(\theta_{i}-b_{j}\right)\right]} \end{array} \end{array}$$

for *i* = 1, …, *N* and *j* = 1, …, *J*, where *N* is the total number of examinees participating in the test and *J* is the test length. Here, *y*_*ij*_ is the binary response of the *i*th examinee with latent ability level θ_*i*_ to answer the *j*th item and is coded as 1 for a correct response and 0 for an incorrect response, *P*_*ij*_ is the corresponding probability of correct response, *a*_*j*_ is the item discrimination parameter, *b*_*j*_ is the item difficulty parameter, *c*_*j*_ is the item lower asymptote (pseudo-guessing) parameter, and *d*_*j*_ is the item upper asymptote parameter. The 4PL model reduces to the other models as special cases: *d*_*j*_ = 1 gives the 3PL model, *c*_*j*_ = 0 gives the 2PL model, and *a*_*j*_ = 1 gives the 1PL model. Following Culpepper (2016), we reparameterize the traditional 4PL model to construct a new 4PL model by defining a slipping parameter similar to that in cognitive diagnostic tests:

(2) $$\begin{array}{l} P_{ij}=P\left(y_{ij}=1|a_{j},b_{j},c_{j},\gamma_{j},\theta_{i}\right)\\ \begin{array}{l} \;=c_{j}+\left(1-\gamma_{j}-c_{j}\right)\frac{\text{exp}\left[1.7a_{j}\left(\theta_{i}-b_{j}\right)\right]}{1+\text{exp}\left[1.7a_{j}\left(\theta_{i}-b_{j}\right)\right]}, \end{array} \end{array}$$

where γ_*j*_ = 1 − *d*_*j*_.

One identification restriction is that the upper asymptote must exceed the lower asymptote: *d*_*j*_ > *c*_*j*_. Equivalently, the restriction 0 < *c*_*j*_ + γ_*j*_ < 1 must be satisfied for the reparameterized 4PL model, Meanwhile, either the scale of latent abilities or the scale of item parameters must be restricted to identify the two0parameter IRT models. Three methods are widely used to identify two-parameter IRT models.

1. Fx the mean population level of ability to zero and the variance population level of ability to one (Lord and Novick, 1968; Bock and Aitkin, 1981; Fox and Glas, 2001; Fox, 2010), i.e., θ ~ *N*(0, 1).
2. Restrict the sum of item difficulty parameters to zero and the product of item discrimination parameters to one (Fox, 2001; Fox, 2005, 2010), i.e., $\overset{J}{\underset{j=1}{\sum }}b_{j}=0$ and $\prod_{j=1}^{J}a_{j}=1$.
3. Fix the item difficulty parameter at a specific value, most often zero, and restrict the discrimination parameter to a specific value, most often one (Fox, 2001; Fox, 2010), i.e., *b*_1_ = 0 and *a*_1_ = 1. The basic idea here is to identify the two-parameter logistic model by anchoring an item discrimination parameter to an arbitrary constant, typically *a*_1_ = 1, for a given item. Meantime, a location identification constraint is imposed by restricting a difficulty parameter, typically *b*_1_ = 0, for a given item. Based on the fixed anchoring values of the item parameters, other parameters are estimated on the same scale. The estimated difficulty or discrimination values of item parameters are interpreted based on their positions relative to the corresponding anchoring values. For details, see Fox (2010, p. 87).

In the present study, the main aim is to evaluate the accuracy of parameter estimation obtained by the slice sampling algorithm for different types of prior distributions. Therefore, the first of the above methods is used to eliminate the trade-offs between ability θ and the difficulty parameter *b* in location, and between ability θ (difficulty parameter *b*) and the discrimination parameter *a* in scale.

## 3. Theoretical Foundation and Analysis of the Advantages of the Slice Sampling Algorithm

### 3.1. Theoretical Foundation of the Slice Sampling Algorithm

The motivation for the slice sampling algorithm (Damien et al., 1999; Neal, 2003; Bishop, 2006; Lu et al., 2018) is that we can use the auxiliary variable approach to sample from posterior distributions arising from Bayesian non-conjugate models. The theoretical basis for this algorithm is as follows.

Suppose that the simulated values are generated from a target density function *t*(*x*) given by *t*(*x*) ∝ $\phi \left(x\right)\prod_{i=1}^{N}$ *l*_*i*_(*x*) that cannot be sampled directly, where ϕ(*x*) is a known density from which samples can be easily drawn and *l*_*i*_(*x*) are non-negative invertible functions, which do not have to be density functions. We introduce the auxiliary variables represented by the vector $\delta ={\left(\delta_{1},\ldots ,\delta_{N}\right)}^{\prime }$, each element of which is from (0, +∞) and where δ_1_, …, δ_*N*_ are mutually independent. The inequalities δ_*i*_ < *l*_*i*_(*x*) are established, and the joint density can be written as

(3) $$\begin{array}{l} t\left(x,\delta_{1},\ldots ,\delta_{N}\right)\propto \phi \left(x\right)\overset{N}{\underset{i=1}{\prod }}\text{I}\left\{\delta_{i}<l_{i}\left(x\right)\right\}, \end{array}$$

where the indicator function I(*A*) takes the value 1 if *A* is true and the value 0 if *A* is false. If the auxiliary variables are integrated out, the marginal distribution *t*(*x*) is obtained as

(4) $$\begin{array}{l} t\left(x\right)=\int_{0}^{l_{1}\left(x\right)}\cdots \int_{0}^{l_{N}\left(x\right)}t\left(x,\delta_{1},\ldots ,\delta_{N}\right)\text{d}\delta_{N}\cdots \text{d}\delta_{1},\\ \begin{array}{l} \;\propto \phi \left(x\right)\int_{0}^{l_{1}\left(x\right)}\cdots \int_{0}^{l_{N}\left(x\right)}1\text{d}\delta_{N}\cdots \text{d}\delta_{1}=\phi \left(x\right)\overset{N}{\underset{i=1}{\prod }}l_{i}\left(x\right). \end{array} \end{array}$$

Using the invertibility of the function *l*_*i*_(*x*), we can then obtain the set Λ_δ_*i*__ = {*x*∣δ_*i*_ < *l*_*i*_(*x*)}. The simulated values are generated from the Gibbs sampler based on the auxiliary variables by repeatedly sampling from the full conditional distributions, proceeding as follows at iteration *r*:

- Sample $\delta_{i}^{\left(r\right)}\sim \text{Uniform}\left(0,l_{i}\left(x^{\left(r-1\right)}\right)\right),$ *i* = 1, …, *N*.
- Sample $x^{\left(r\right)}\sim \Lambda_{\delta_{i}}=\left\{x|\delta_{i}^{\left(r\right)}<l_{i}\left(x\right)\right\}$.

We thereby derive a horizontal “slice” under the density function. Thus, a Markov chain based on the new Gibbs sampler can be constructed by sampling points alternately from the uniform distribution under the density curve and only concerning the horizontal “slice” defined by the current sample points.

### 3.2. Advantages of the Slice Sampling Algorithm Compared With the Metropolis–Hastings Algorithm

In the Bayesian framework, we first consider the benefits of the slice sampling algorithm compared with the traditional Metropolis–Hastings (MH) algorithm (Metropolis et al., 1953; Hastings, 1970; Tierney, 1994; Chib and Greenberg, 1995; Chen et al., 2000). It is known that the MH algorithm relies heavily on the tuning parameters of the proposal distribution for different data sets. In addition, the MH algorithm is sensitive to step size. If the step size is too small, the chain will take longer to traverse the target density. If the step size is too large, there will be inefficiencies due to high rejection rate. More specifically, researchers should ensure that each parameter candidate is no more than 50% accepted by adjusting the tuning parameters of the MH algorithm. Further, for example, when we draw two-dimensional item parameters at the same time in the 2PL model, the probability of acceptance will be reduced to around 25% (Patz and Junker, 1999, p. 163). Thus, the sampling efficiency of the MH algorithm is greatly reduced. However, the slice sampling algorithm avoids the retrospective tuning that is needed in the MH algorithm if we do not know how to choose a proper tuning parameter or if no value for the tuning parameter is appropriate. It always keeps the drawn samples accepted, thus increasing the sampling efficiency. Next, we show that the slice sampling algorithm is more efficient than a particular independent MH chain.

Let us use the MH algorithm to obtain samples from the posterior distribution *t*(*x*) given by *t*(*x*) ∝ ϕ(*x*)*l*(*x*), where ϕ(*x*) is selected as a special proposal distribution. Let x^*^ be a candidate value from the proposal distribution ϕ(*x*) and let *x*^(*r*)^ be the current point. The probability of the new candidate being accepted, min{1, *l*(x^*^)/*l*(*x*^(*r*)^)}, is determined by a random number *u* from Uniform(0, 1). Essentially, if *u* < *l*(x^*^)/*l*(*x*^(*r*)^), then *x*^(*r*+1)^ = x^*^; otherwise, *x*^(*r*+1)^ = *x*^(*r*)^. The process is to draw the candidate first and then determine whether or not to “move” or “stay” by using the random number *u*. The “stay” process will lead to a reduction in the sampling efficiency of the MH algorithm. By contrast, suppose we consider the inverse process of the above sampling to draw the random number *u* first. To achieve the purpose of moving, we need to draw the candidate x^*^ from ϕ(*x*) such that *u* < *l*(x^*^)/*l*(*x*^(*r*)^). Therefore, x^*^ can be regarded as a sample from ϕ(*x*) restricted to the set $\Theta_{u}\left(r\right)=\left\{x|l\left(x\right)>ul\left(x^{\left(r\right)}\right)\right\}$. In this case, the chain will always be moved, thus improving the sampling efficiency.

In addition, with the MH algorithm, it is relatively difficult to sample parameters with monotonicity or truncated interval restrictions. Instead, it is possible to improve the accuracy of parameter estimation by employing strong informative prior distributions to avoid violating the restriction conditions (Culpepper, 2016). For example, the prior distributions of the lower asymptote and upper asymptote parameters used in Loken and Rulison (2010) are, respectively Beta(5, 17) and Beta(17, 5), and these two parameters are fairly concentrated in the range of 0.227–0.773. However, the advantage of the slice sampling algorithm is that it can easily draw the posterior samples from any prior distribution as long as these distributions have a reasonable value range of parameters. See the following sections for details.

### 3.3. Advantages of the Slice Sampling Algorithm Compared With the Gibbs Algorithm

The idea of the slice sampling algorithm is to draw the posterior samples from a truncated prior distribution by introducing auxiliary variables, where the truncated interval is deduced from the likelihood function. This differs from the approach of the Gibbs algorithm (Geman and Geman, 1984; Gelfand and Smith, 1990), which is to generate posterior samples by sweeping through each variable to sample from its conditional distribution, with the remaining variables fixed at their current values. However, slice sampling algorithm can be conceived of as extensions of the Gibbs algorithm. In particular, when the parameters in which we are interested are represented by a multidimensional vector ***X***, we cannot use the slice sampling algorithm directly to obtain the multivariate set $\Theta_{u}=\left(\Theta_{u}^{1},\ldots ,\Theta_{u}^{k},\ldots ,\Theta_{u}^{p}\right)$, where *p* is the dimension of ***X***. Therefore, a Gibbs sampler is employed to draw the samples from the full conditional distribution *l*(*x*_*k*_∣***x***_(−*k*)_, *u*) for *k* = 1, …, *p*, which is a realization of *t*(***X***). This involves sampling from ϕ(*x*_*k*_∣***x***_(−*k*)_) restricted to the set $\Theta_{u}^{k}=\left\{x_{k}|l\left(x_{k},x_{\left(-k\right)}\right)>u\right\}$, where the premise must be satisfied that *l*(*x*_*k*_, ***x***_(−*k*)_) is invertible for all *k* given ***x***_(−*k*)_.

It is well-known that the Gibbs algorithm can quickly and effectively draw samples from the posterior distribution owing to the fact that the full conditional posterior distribution is easy to sample using the conjugate prior distribution. However, the Gibbs algorithm is not valid for Bayesian non-conjugate models such as the 2PL model. By comparison, the slice sampling algorithm for estimating the 2PL model has the advantage of a flexible prior distribution being introduced to obtain samples from the full conditional posterior distributions rather than being restricted to using the conjugate distributions, which is required in Gibbs sampling and is limited to the use of the normal ogive framework (Tanner and Wong, 1987; Albert, 1992; Béguin and Glas, 2001; Fox and Glas, 2001; Fox, 2010; Culpepper, 2016). The slice sampling algorithm allows the use of informative prior distributions and non-informative prior distributions, and even if an inappropriate prior distribution is adopted, it can still obtain satisfactory results. That is, any prior distribution can be used as long as the values sampled from it are in a reasonable range of the parameter support set. For example, for the discrimination parameter, the following prior distributions can be considered: the informative prior log*N*(0, 1), the non-informative priors *N*(0, 1000)I(*a* > 0), and the inappropriate priors Exp(1) and Gamma(2, 3).

## 4. Bayesian Inference

### 4.1. Bayesian Estimation

In the present study, an efficient Gibbs-slice sampling algorithm in a fully Bayesian framework is used to estimate the following 4PL model. The sampling process of Gibbs-slice sampling algorithm consists of two parts. One part is the Gibbs sampling algorithm, which is used to update the guessing and slipping parameters from the truncated Beta distributions by introducing auxiliary variables (Béguin and Glas, 2001; Fox, 2010; Culpepper, 2016). The efficiency of Gibbs sampling is greatly improved by the use of conjugate prior distributions (Tanner and Wong, 1987; Albert, 1992). The other part is the slice sampling algorithm, which samples the 2PL model from the truncated full conditional posterior distributions by introducing different auxiliary variables.

Next, the specific sampling process of the Gibbs-slice sampling algorithm is described.

###  Gibbs Steps

First, following Béguin and Glas (2001), we introduce an auxiliary variable η_*ij*_, where η_*ij*_ = 1 indicates that examinee *i* has the ability to answer item *j* correctly and η_*ij*_ = 0 otherwise. The purpose of introducing this auxiliary variable is to separate the guessing and slipping parameters from the 4PL model and make it easier to implement Gibbs sampling for the guessing and slipping parameters through the conjugate Beta distributions. Letting Δ = (θ_*i*_, *a*_*j*_, *b*_*j*_, *c*_*j*_, γ_*j*_), we can obtain the full conditional distribution of η_*ij*_ based on Bayes' theorem:

(5) $$\begin{array}{l} P(\eta_{ij}=1|y_{ij}=1,\Delta )=\frac{P(\eta_{ij}=1,y_{ij}=1,\Delta )}{P(y_{ij}=1|\Delta )}\\ \;=\frac{(1-\gamma_{j})P_{ij}^{*}}{c_{j}+(1-\gamma_{j}-c_{j})P_{ij}^{*}},\\ P(\eta_{ij}=0|y_{ij}=1,\Delta )=\frac{P(\eta_{ij}=0,y_{ij}=1,\Delta )}{P(y_{ij}=1|\Delta )}\\ \;=\frac{c_{j}(1-P_{ij}^{*})}{c_{j}+(1-\gamma_{j}-c_{j})P_{ij}^{*}},\\ P(\eta_{ij}=1|y_{ij}=0,\Delta )=\frac{P(\eta_{ij}=1,y_{ij}=0,\Delta )}{P(y_{ij}=0|\Delta )}\\ \;=\frac{\gamma_{j}P_{ij}^{*}}{\;1-c_{j}-(1-\gamma_{j}-c_{j})P_{ij}^{*}},\\ P(\eta_{ij}=0|y_{ij}=0,\Delta )=\frac{P(\eta_{ij}=0,y_{ij}=0,\Delta )}{P(y_{ij}\%=0|\Delta )}\\ \;=\frac{\left(1-c_{j})(1-P_{ij}^{*}\right)}{1-c_{j}-(1-\gamma_{j}-c_{j})P_{ij}^{*}}. \end{array}$$

where

$$P_{ij}^{*}=\frac{\text{exp}\left[1.7a_{j}\left(\theta_{i}-b_{j}\right)\right]}{1+\text{exp}\left[1.7a_{j}\left(\theta_{i}-b_{j}\right)\right]}.$$

The priors of the guessing and slipping parameters follow the Beta distributions, i.e., *c*_*j*_ ~ Beta(ν_0_, *u*_0_), γ_*j*_ ~ Beta(ν_1_, *u*_1_). However, the guessing and slipping parameters themselves satisfy the following truncated restrictions owing to model identification (Junker and Sijtsma, 2001; Culpepper, 2016):

(6) $$\begin{array}{l} \Xi =\left\{\left(c_{j},\gamma_{j}\right)|0\leq c_{j}<1,0\leq \gamma_{j}<1,0\leq c_{j}<1-\gamma_{j}\right\}. \end{array}$$

The joint posterior distribution of the guessing and slipping parameters can be written as

(7) $$\begin{array}{l} p\left(c_{j},\gamma_{j}|y_{j},\eta_{j}\right)\propto \overset{N}{\underset{i=1}{\prod }}{\left[{\left(1-\gamma_{j}\right)}^{\eta_{ij}}c_{j}^{\left(1-\eta_{ij}\right)}\right]}^{y_{ij}}{\left[\gamma_{j}^{\eta_{ij}}{\left(1-c_{j}\right)}^{\left(1-\eta_{ij}\right)}\right]}^{\left(1-y_{ij}\right)}\\ \;p\left(c_{j},\gamma_{j}\right)\text{I}(\left(c_{j},\gamma_{j}\right)\in \Xi )\propto c_{j}^{{\hat{\kappa }}_{00}+\nu_{0}-1}{\left(1-c_{j}\right)}^{{\hat{\kappa }}_{01}+u_{0}-1}\\ \;\gamma_{j}^{{\hat{\kappa }}_{10}+\nu_{1}-1}{\left(1-\gamma_{j}\right)}^{{\hat{\kappa }}_{11}+u_{1}-1}\text{I}(\left(c_{j},\gamma_{j}\right)\in \Xi ). \end{array}$$

Let $y_{j}^{\prime }=\left(y_{1j},\ldots ,y_{Nj}\right)$, $\eta_{j}^{\prime }=\left(\eta_{1j},\ldots ,\eta_{Nj}\right)$, and

$$\begin{array}{l} {\hat{\kappa }}_{00}={\left(1_{N}-\eta_{j}\right)}^{\prime }y_{j},\;{\hat{\kappa }}_{01}={\left(1_{N}-\eta_{j}\right)}^{\prime }\left(1_{N}-y_{j}\right),\;\\ {\hat{\kappa }}_{10}=\eta_{j}^{\prime }\left(1_{N}-y_{j}\right),\;{\hat{\kappa }}_{11}=\eta_{j}^{\prime }y_{j}. \end{array}$$

The full conditional posterior distributions of (*c*_*j*_, γ_*j*_) can be written as

(8) $$\begin{array}{l} c_{j}^{\left(r\right)}|\gamma_{j}^{\left(r-1\right)}\sim \text{Beta}\left({\hat{\kappa }}_{00}+\nu_{0},{\hat{\kappa }}_{01}+u_{0}\right)\text{I}\left(0\leq c_{j}^{\left(r\right)}<1-\gamma_{j}^{\left(r-1\right)}\right),\\ \begin{array}{l} \gamma_{j}^{\left(r\right)}|c_{j}^{\left(r\right)}\sim \text{Beta}\left({\hat{\kappa }}_{10}+\nu_{1},{\hat{\kappa }}_{11}+u_{1}\right)\text{I}\left(0\leq \gamma_{j}^{\left(r\right)}<1-c_{j}^{\left(r\right)}\right). \end{array} \end{array}$$

###  Slice Steps

Supposing that the guessing and slipping parameters have been updated by the Gibbs algorithm, we update the parameters in the 2PL model using the slice sampling algorithm. Two additional independent auxiliary variables λ_*ij*_ and φ_*ij*_, defined on the intervals

$$\left(0,\frac{P_{ij}^{\left(r\right)}-c_{j}^{\left(r\right)}}{1-\gamma_{j}^{\left(r\right)}-c_{j}^{\left(r\right)}}\right)\text{ and }\left(0,\frac{1-\gamma_{j}^{\left(r\right)}-P_{ij}^{\left(r\right)}}{1-\gamma_{j}^{\left(r\right)}-c_{j}^{\left(r\right)}}\right),$$

are introduced to facilitate sampling, where *r* is the number of iterations. In fact, (*P*_*ij*_ − *c*_*j*_)/(1 − γ_*j*_ − *c*_*j*_) is the correct response probability of the 2PL model, while (1 − γ_*j*_ − *P*_*ij*_)/(1 − γ_*j*_ − *c*_*j*_) is the corresponding incorrect response probability. Therefore, the joint likelihood of *a, b, c*, γ, θ based on the auxiliary variables λ and φ can be written as

(9) $$\begin{array}{l} p(y|a,b,\theta ,c,\gamma ,\lambda ,\varphi )\\ \;\propto \prod_{i\;=\;1}^{N}\prod_{j\;=\;1}^{J}[\text{I}(y_{ij}=1)\text{I}\left(0<\lambda_{ij}\leq \frac{P_{ij}-c_{j}}{1-\gamma_{j}-c_{j}}\right)\\ \;+\text{I}(y_{ij}=0)\text{I}\left(0<\varphi_{ij}\leq \frac{1-\gamma_{j}-P_{ij}}{1-\gamma_{j}-c_{j}}\right)]. \end{array}$$

Equivalently,

(10) $$\begin{array}{l} p\left(y|a,b,\theta ,c,\gamma ,\lambda ,\varphi \right)\propto \prod_{i=1}^{N}\prod_{j=1}^{J}\text{I}\left(y_{ij}=1\right)\text{I}\left(0<\lambda_{ij}\leq P_{ij}^{*}\right)\\ \begin{array}{l} \;+\text{I}\left(y_{ij}=0\right)\text{I}\left(0<\varphi_{ij}\leq Q_{ij}^{*}\right), \end{array} \end{array}$$

where

$$\begin{array}{l} P_{ij}^{*}=1-Q_{ij}^{*}=\frac{\text{exp}\left[1.7a_{j}\left(\theta_{i}-b_{j}\right)\right]}{1+\text{exp}\left[1.7a_{j}\left(\theta_{i}-b_{j}\right)\right]}=\frac{P_{ij}-c_{j}}{1-\gamma_{j}-c_{j}},\\ Q_{ij}^{*}=\frac{1}{1+\text{exp}\left[1.7a_{j}\left(\theta_{i}-b_{j}\right)\right]}=\frac{1-\gamma_{j}-P_{ij}}{1-\gamma_{j}-c_{j}}. \end{array}$$

Integrating out the two random variables λ and φ in (10), the joint likelihood based on responses can be obtained:

(11) $$\begin{array}{l} p\left(y|a,b,\theta ,c,\gamma ,\lambda ,\varphi \right)\propto \overset{N}{\underset{i=1}{\prod }}\overset{J}{\underset{j=1}{\prod }}\text{I}\left(y_{ij}=1\right)E_{\lambda }\left[\text{I}\left(0<\lambda_{ij}\leq P_{ij}^{*}\right)\right]\\ \;+\text{I}\left(y_{ij}=0\right)E_{\varphi }\left[\text{I}\left(0<\varphi_{ij}\leq Q_{ij}^{*}\right)\right]\\ \begin{array}{l} \;\propto \overset{N}{\underset{i=1}{\prod }}\overset{J}{\underset{j=1}{\prod }}{\left(P_{ij}^{*}\right)}^{\left(y_{ij}=1\right)}{\left(Q_{ij}^{*}\right)}^{\left(y_{ij}=0\right)}, \end{array} \end{array}$$

where *E*_λ_ is an expectation operation for the random variable λ. We know that **η**, **λ**, and ***φ*** are independent of each other. Therefore, the joint posterior distribution based on the auxiliary variables can be written as

(12) $$\begin{array}{l} p\left(\eta ,\theta ,a,b,c,\gamma ,\lambda ,\varphi |y\right)\propto p\left(\eta |a,b,\theta ,c,\gamma ,y\right)p\left(\lambda ,\varphi |a,b,\theta ,c,\gamma ,y\right)\\ \begin{array}{l} \;\times p\left(\theta \right)p\left(a\right)p\left(b\right)p\left(c,\gamma \right)\text{I}(\left(c,\gamma \right)\in \Xi ). \end{array} \end{array}$$

The specific form can be represented as

(13) $$\begin{array}{l} p(\eta ,a,b,\theta ,c,\gamma ,\lambda ,\varphi |y)\propto \prod_{i\;=\;1}^{N}\prod_{j\;=\;1}^{J}{\left[{\left(1-\gamma_{j}\right)}^{\eta_{ij}}c_{j}^{\left(1-\eta_{ij}\right)}\right]}^{y_{ij}}\\ \;{\left[\gamma_{j}^{\eta_{ij}}{\left(1-c_{j}\right)}^{\left(1-\eta_{ij}\right)}\right]}^{\left(1-y_{ij}\right)}\\ \;\times [\text{I}(y_{ij}=1)\text{I}(0<\lambda_{ij}\leq P_{ij}^{*})\\ \;+\text{I}(y_{ij}=0)\text{I}(0<\varphi_{ij}\leq Q_{ij}^{*})]\\ \;\times \prod_{j\;=\;1}^{J}\left(a_{j})p(b_{j})p(c_{j},\gamma_{j})\text{I}((c_{j},\gamma_{j})\in \Xi \right)\\ \;\prod_{i\;=\;1}^{N}p(\theta_{i}). \end{array}$$

The detailed slice sampling algorithm is given below.

First, we update the auxiliary variables λ_*ij*_ and φ_*ij*_ when given θ_*i*_, *a*_*j*_, *b*_*j*_, *c*_*j*_, γ_*j*_, and *y*_*ij*_. According to (13), the auxiliary variables λ_*ij*_ and φ_*ij*_ have the following interval constraints:

$$\begin{array}{l} 0<\lambda_{ij}\leq P_{ij}^{*}=\frac{P_{ij}-c_{j}}{1-\gamma_{j}-c_{j}}\text{ when }y_{ij}=1,\\ 0<\varphi_{ij}\leq Q_{ij}^{*}=\frac{1-\gamma_{j}-P_{ij}}{1-\gamma_{j}-c_{j}}\text{ when }y_{ij}=0. \end{array}$$

Therefore, the full conditional posterior distributions of λ_*ij*_ and φ_*ij*_ can be written as

(14) $$\begin{array}{l} \lambda_{ij}|\theta_{i},a_{j},b_{j},c_{j},\gamma_{j},y_{ij}\sim \text{Uniform}\left(0,\frac{P_{ij}-c_{j}}{1-\gamma_{j}-c_{j}}\right)\text{ when}\\ \begin{array}{l} {\;y}_{ij}=1, \end{array} \end{array}$$

(15) $$\begin{array}{l} \varphi_{ij}|\theta_{i},a_{j},b_{j},c_{j},\gamma_{j},y_{ij}\sim \text{Uniform}\left(0,\frac{1-\gamma_{j}-P_{ij}}{1-\gamma_{j}-c_{j}}\right)\text{ when}\\ \begin{array}{l} {\;y}_{ij}=0. \end{array} \end{array}$$

Next, we update the difficulty parameter *b*_*j*_. The prior of the difficulty parameter is assumed to follow a normal distribution with mean μ_*b*_ and variance $\sigma_{b}^{2}$. According to (10), ∀*i*, when *y*_*ij*_ = 1, we have $0<\lambda_{ij}\leq P_{ij}^{*}$, and the following inequality can be established:

$$\begin{array}{l} a_{j}\left(\theta_{i}-b_{j}\right)\geq \frac{1}{1.7}\text{log}\left(\frac{\lambda_{ij}}{1-\lambda_{ij}}\right),\text{or equivalently}\\ {\;b}_{j}\leq \theta_{i}-\frac{1}{1.7a_{j}}\text{log}\left(\frac{\lambda_{ij}}{1-\lambda_{ij}}\right). \end{array}$$

In fact, this inequality is obtained through the following calculation process:

$$0<\lambda_{ij}\leq P_{ij}^{*},\text{or equivalently }0<\lambda_{ij}\leq \frac{\text{exp}\left[1.7a_{j}\left(\theta_{i}-b_{j}\right)\right]}{1+\text{exp}\left[1.7a_{j}\left(\theta_{i}-b_{j}\right)\right]},$$

from which

$$\begin{array}{l} \lambda_{ij}+\lambda_{ij}\text{exp}\left[1.7a_{j}\left(\theta_{i}-b_{j}\right)\right]\leq \text{exp}\left[1.7a_{j}\left(\theta_{i}-b_{j}\right)\right],\text{or equivalently}\\ \;\frac{\lambda_{ij}}{1-\lambda_{ij}}\leq \text{exp}\left[1.7a_{j}\left(\theta_{i}-b_{j}\right)\right]. \end{array}$$

Therefore, we have

$$\begin{array}{l} \text{log}\left(\frac{\lambda_{ij}}{1-\lambda_{ij}}\right)\leq \left[1.7a_{j}\left(\theta_{i}-b_{j}\right)\right],\text{or equivalently}\\ {\;a}_{j}\left(\theta_{i}-b_{j}\right)\geq \frac{1}{1.7}\text{log}\left(\frac{\lambda_{ij}}{1-\lambda_{ij}}\right). \end{array}$$

Finally, we obtain the following inequality:

$$b_{j}\leq \theta_{i}-\frac{1}{1.7a_{j}}\text{log}\left(\frac{\lambda_{ij}}{1-\lambda_{ij}}\right).$$

In the same way, ∀*i*, when *y*_*ij*_ = 0, we have $0<\varphi_{ij}\leq Q_{ij}^{*}$. Therefore, the following inequality can be obtained:

$$\begin{array}{l} a_{j}\left(\theta_{i}-b_{j}\right)\leq \frac{1}{1.7}\text{log}\left(\frac{1-\varphi_{ij}}{\varphi_{ij}}\right),\text{or equivalently}\\ {\;b}_{j}\geq \theta_{i}-\frac{1}{1.7a_{j}}\text{log}\left(\frac{1-\varphi_{ij}}{\varphi_{ij}}\right). \end{array}$$

Using the above inequalities $0<\lambda_{ij}\leq P_{ij}^{*}$ and $0<\varphi_{ij}\leq Q_{ij}^{*})$, we can obtain a truncated interval about the difficulty parameter *b*_*j*_:

$$\theta_{i}-\frac{1}{1.7a_{j}}\text{log}\left(\frac{1-\varphi_{ij}}{\varphi_{ij}}\right)\leq b_{j}\leq \theta_{i}-\frac{1}{1.7a_{j}}\text{log}\left(\frac{\lambda_{ij}}{1-\lambda_{ij}}\right).$$

If this truncated interval is narrow, the sampling efficiency is improved and the parameter can converge fast. Therefore, we need to limit the upper and lower bounds of the truncated interval. In fact, we can obtain a maximum of θ_*i*_ − (1/1.7*a*_*j*_)log[(1 − φ_*ij*_)/φ_*ij*_] among all the examinees who correctly answer the *j*th item. Similarly, we can obtain a minimum of θ_*i*_ − (1/1.7*a*_*j*_)log[λ_*ij*_/(1 − λ_*ij*_)] among all the examinees who mistakenly answer the *j*th item. Finally, the full conditional posterior distribution of *b*_*j*_ can be obtained as a truncated prior distribution, with the truncated interval between maximum and minimum. The specific mathematical expressions are as follows.

Let $D_{j}=\left\{i|y_{ij}=1,0<\lambda_{ij}\leq P_{ij}^{*}\right\}$ and $F_{j}=\left\{i|y_{ij}=0,0<\varphi_{ij}\leq Q_{ij}^{*}\right\}$. Then, given *a*_*j*_, *c*_*j*_, γ_*j*_, ***θ***, **λ**, ***φ***, and ***y***, the full conditional posterior distribution of *b*_*j*_ is

(16) $$\begin{array}{l} b_{j}|a_{j},c_{j},\gamma_{j},\theta ,\lambda ,\varphi ,y\sim N\left(\mu_{b},\sigma_{b}^{2}\right)\text{I}\left(b_{j}^{L}\leq b_{j}\leq b_{j}^{U}\right), \end{array}$$

where

$$\begin{array}{l} b_{j}^{L}=\underset{i\in F_{j}}{\text{max}}\left\{\theta_{i}-\frac{1}{1.7a_{j}}\text{log}\left(\frac{1-\varphi_{ij}}{\varphi_{ij}}\right)\right\}\text{ and}\\ \;b_{j}^{U}=\underset{i\in D_{j}}{\text{min}}\left\{\theta_{i}-\frac{1}{1.7a_{j}}\text{log}\left(\frac{\lambda_{ij}}{1-\lambda_{ij}}\right)\right\}. \end{array}$$

Subsequently, we update the discrimination parameter *a*_*j*_. To ensure that this parameter is greater than zero, we use a truncated normal distribution with mean μ_*a*_ and variance $\sigma_{a}^{2}$ as a prior distribution, $N\left(\mu_{a},\sigma_{a}^{2}\right)\text{I}\left(a_{j}>0\right)$. Under the condition *y*_*ij*_ = 1, ∀*i*, θ_*i*_ − *b*_*j*_ > 0, we have $0<\lambda_{ij}\leq P_{ij}^{*}$, while under the condition *y*_*ij*_ = 0, ∀*i*, θ_*i*_ − *b*_*j*_ < 0, we have $0<\varphi_{ij}\leq Q_{ij}^{*}$. The following inequalities concerning the discrimination parameter *a*_*j*_ can be established using a procedure similar to that used above to derive the truncated interval for the difficulty parameter *b*_*j*_:

$$\begin{array}{l} a_{j}\geq \frac{1}{1.7\left(\theta_{i}-b_{j}\right)}\text{log}\left(\frac{\lambda_{ij}}{1-\lambda_{ij}}\right),\\ a_{j}\geq \frac{1}{1.7\left(\theta_{i}-b_{j}\right)}\text{log}\left(\frac{1-\varphi_{ij}}{\varphi_{ij}}\right). \end{array}$$

Similarly, when *y*_*ij*_ = 1, ∀*i*, θ_*i*_ − *b*_*j*_ < 0, we have $0<\lambda_{ij}\leq P_{ij}^{*}$, and when *y*_*ij*_ = 0, ∀*i*, θ_*i*_ − *b*_*j*_ > 0, we have $0<\varphi_{ij}\leq Q_{ij}^{*}$, from which we obtain

$$\begin{array}{l} a_{j}\leq \frac{1}{1.7\left(\theta_{i}-b_{j}\right)}\text{log}\left(\frac{\lambda_{ij}}{1-\lambda_{ij}}\right)\\ a_{j}\leq \frac{1}{1.7\left(\theta_{i}-b_{j}\right)}\text{log}\left(\frac{1-\varphi_{ij}}{\varphi_{ij}}\right). \end{array}$$

Let

$$\begin{array}{l} \Delta_{j}=\left\{i|y_{ij}=1,\theta_{i}-b_{j}>0,0<\lambda_{ij}\leq P_{ij}^{*}\right\},\\ H_{j}=\left\{i|y_{ij}=0,\theta_{i}-b_{j}<0,0<\varphi_{ij}\leq Q_{ij}^{*}\right\},\\ {▽}_{j}=\left\{i|y_{ij}=1,\theta_{i}-b_{j}<0,0<\lambda_{ij}\leq P_{ij}^{*}\right\},\\ \Lambda_{j}=\left\{i|y_{ij}=0,\theta_{i}-b_{j}>0,0<\varphi_{ij}\leq Q_{ij}^{*}\right\}. \end{array}$$

Given *b*_*j*_, *c*_*j*_, γ_*j*_, **λ**, ***φ***, ***θ***, and ***y***, the full conditional posterior distribution of *a*_*j*_ is given by

(17) $$\begin{array}{l} a_{j}|b_{j},c_{j},\gamma_{j},\lambda ,\varphi ,\theta ,y\sim N\left(\mu_{a},\sigma_{a}^{2}\right)\text{I}\left(0<a_{j}^{L}\leq a_{j}\leq a_{j}^{U}\right), \end{array}$$

where

$$\begin{array}{l} a_{j}^{L}=\text{max}\{0,\underset{i\in \Delta_{j}}{\text{max}}\left\{\frac{1}{1.7(\theta_{i}-b_{j})}\text{log}\left(\frac{\lambda_{ij}}{1-\lambda_{ij}}\right)\right\},\\ \;\underset{i\in H_{j}}{\text{max}}\left\{\frac{1}{1.7(\theta_{i}-b_{j})}\text{log}\left(\frac{1-\varphi_{ij}}{\varphi_{ij}}\right)\right\}\},\\ a_{j}^{U}=\text{min}\{\underset{{\;}_{i\in {▽}_{j}}}{\text{min}}\left\{\frac{1}{1.7(\theta_{i}-b_{j})}\text{log}\left(\frac{\lambda_{ij}}{1-\lambda_{ij}}\right)\right\},\\ \;\underset{i\in \Lambda_{j}}{\text{min}}\left\{\frac{1}{1.7(\theta_{i}-b_{j})}\log\left(\frac{1-\varphi_{ij}}{\varphi_{ij}}\right)\right\}\}. \end{array}$$

In fact, the discrimination parameter is set to be greater than zero in the item response theory. Therefore, the prior distribution for the discrimination parameter is assumed to be a normal distribution truncated at 0. Based on the likelihood information, we can obtain the truncation interval of the discrimination parameter. However, the left endpoint of the truncation interval may be <0. In this case, we need to add 0 to the truncation interval to restrict the left endpoint in 17.

Finally, we update the latent ability θ_*i*_. The prior of θ_*i*_ is assumed to follow a normal distribution, $\theta_{i}\sim N\left(\mu_{\theta },\sigma_{\theta }^{2}\right)$. The latent ability θ_*i*_ is sampled from the following normal distribution with truncated interval between $\theta_{i}^{L}$ and $\theta_{i}^{U}$:

(18) $$\begin{array}{l} \theta_{i}|\lambda ,\varphi ,a,b,c,\gamma ,y\sim N\left(\mu_{\theta },\sigma_{\theta }^{2}\right)\text{I}\left(\theta_{i}^{L}\leq \theta_{i}\leq \theta_{i}^{U}\right), \end{array}$$

where

$$\begin{array}{l} \theta_{i}^{L}=\underset{j\in D_{i}}{\text{max}}\left\{\frac{1}{1.7a_{j}}\text{log}\left(\frac{\lambda_{ij}}{1-\lambda_{ij}}\right)+b_{j}\right\},\\ \theta_{i}^{U}=\underset{j\in F_{i}}{\text{min}}\left\{\frac{1}{1.7a_{j}}\text{log}\left(\frac{1-\varphi_{ij}}{\varphi_{ij}}\right)+b_{j}\right\}. \end{array}$$

### 4.2. Bayesian Model Assessment

In this paper, two Bayesian model assessment methods are considered to fit three different models (the 2PL, 3PL, and 4PL models), namely, the deviance information criterion (DIC; Spiegelhalter et al., 2002) and the logarithm of the pseudomarginal likelihood (LPML; Geisser and Eddy, 1979; Ibrahim et al., 2001). These two criteria are based on the log-likelihood functions evaluated at the posterior samples of the model parameters. Therefore, the DIC and LPML of the 4PL model can be easily computed. Write **Ω** = (**Ω**_*ij*_, *i* = 1, …, *N, j* = 1, …, *J*), where $\Omega_{ij}={\left(\theta_{i},a_{j},b_{j},c_{j},\gamma_{j}\right)}^{\prime }$. Let {**Ω**^(1)^, …, **Ω**^(*R*)^} denote an MCMC sample from the full conditional posterior distribution in (8) and (16)–(18), where $\Omega^{\left(r\right)}=\left(\Omega_{ij}^{\left(r\right)},i=1,\ldots ,N,\;j=1,\ldots ,J\right)$ and $\Omega_{ij}^{\left(r\right)}={\left(\theta_{i}^{\left(r\right)},a_{j}^{\left(r\right)},b_{j}^{\left(r\right)},c_{j}^{\left(r\right)},\gamma_{j}^{\left(r\right)}\right)}^{\prime }$ for *i* = 1, …, *N*, *j* = 1, …, *J*, and *r* = 1, …, *R*. The joint likelihood function of the responses can be written as

(19) $$\begin{array}{l} L\left(Y|\Omega \right)=\overset{N}{\underset{i=1}{\prod }}\overset{J}{\underset{j=1}{\prod }}f\left(y_{ij}|\theta_{i},a_{j},b_{j},c_{j},\gamma_{j}\right), \end{array}$$

where *f*(*y*_*ij*_∣θ_*i*_, *a*_*j*_, *b*_*j*_, *c*_*j*_, γ_*j*_) is the probability of response. The logarithm of the joint likelihood function in (19) evaluated at **Ω**^(*r*)^ is given by

(20) $$\begin{array}{l} \text{log}L\left(Y|\Omega^{\left(r\right)}\right)=\overset{N}{\underset{i=1}{\sum }}\overset{J}{\underset{j=1}{\sum }}\text{log}f\left(y_{ij}|\theta_{i}^{\left(r\right)},a_{j}^{\left(r\right)},b_{j}^{\left(r\right)},c_{j}^{\left(r\right)},\gamma_{j}^{\left(r\right)}\right). \end{array}$$

Since the joint log-likelihoods for the responses, $\text{log}f\left(y_{ij}|\theta_{i}^{\left(r\right)},a_{j}^{\left(r\right)},b_{j}^{\left(r\right)},c_{j}^{\left(r\right)},\gamma_{j}^{\left(r\right)}\right)$, *i* = 1, …, *N* and *j* = 1, …, *J*, are readily available from MCMC sampling outputs, $\text{log}f\left(y_{ij}|\theta_{i}^{\left(r\right)},a_{j}^{\left(r\right)},b_{j}^{\left(r\right)},c_{j}^{\left(r\right)},\gamma_{j}^{\left(r\right)}\right)$ in (20) is easy to compute. We now calculate DIC as follows:

(21) $$\begin{array}{l} \text{DIC}=\hat{\text{Dev}\left(\Omega \right)}+2P_{D}=\hat{\text{Dev}\left(\Omega \right)}+2\left[\bar{\text{Dev}\left(\Omega \right)}-\hat{\text{Dev}\left(\Omega \right)}\right], \end{array}$$

where

$$\begin{array}{l} \bar{\text{Dev}\left(\Omega \right)}=-\frac{2}{R}\overset{R}{\underset{r=1}{\sum }}\text{log }L\left(Y|\Omega^{\left(r\right)}\right)\text{ and}\\ \;\hat{\text{Dev}\left(\Omega \right)}=-2\underset{1\leq r\leq R}{\text{max}}\text{log }L\left(Y|\Omega^{\left(r\right)}\right). \end{array}$$

In (21), $\bar{\text{Dev}\left(\Omega \right)}$ is a Monte Carlo estimate of the posterior expectation of the deviance function Dev(**Ω**) = −2log *L*(***Y*** ∣ **Ω**), $\hat{\text{Dev}\left(\Omega \right)}$ is an approximation of $\text{Dev}\left(\hat{\Omega }\right)$, where $\hat{\Omega }$ is the posterior mode, when the prior is relatively non-informative, and $P_{D}=\bar{\text{Dev}\left(\Omega \right)}-\hat{\text{Dev}\left(\Omega \right)}$ is the effective number of parameters. Based on our construction, both DIC and *P*_*D*_ given in (21) are always non-negative. The model with a smaller DIC value fits the data better.

Letting $U_{ij,\text{max}}=\text{max }1\leq r\leq R\left\{-\text{log }f\left(y_{ij}|\theta_{i}^{\left(r\right)},a_{j}^{\left(r\right)},b_{j}^{\left(r\right)},c_{j}^{\left(r\right)},\gamma_{j}^{\left(r\right)}\right)\right\}$, we obtain a Monte Carlo estimate of the conditional predictive ordinate (CPO; Gelfand et al., 1992; Chen et al., 2000) as

(22) $$\begin{array}{l} \text{log}\hat{\left({\text{CPO}}_{ij}\right)}=-U_{ij,\text{max}}-\text{log}\{\frac{1}{R}\sum_{r\;=\;1}^{R}\text{exp}[\\ \;-\text{log }f(y_{ij}|\theta_{i}^{\left(r\right)},a_{j}^{\left(r\right)},b_{j}^{\left(r\right)},c_{j}^{\left(r\right)},\gamma_{j}^{\left(r\right)})-U_{ij,\text{max}}]\}. \end{array}$$

Note that the maximum value adjustment used in $\text{log}\hat{\left(\text{CP}{\text{O}}_{ij}\right)}$ plays an important role in numerical stabilization in computing $\text{exp}\left[-\text{log }f\left(y_{ij}|\theta_{i}^{\left(r\right)},a_{j}^{\left(r\right)},b_{j}^{\left(r\right)},c_{j}^{\left(r\right)},\gamma_{j}^{\left(r\right)}\right)-U_{ij,\text{max}}\right]$ in (22). A summary statistic of the $\hat{\text{CP}{\text{O}}_{ij}}$ is the sum of their logarithms, which is called the LPML and given by

(23) $$\begin{array}{l} \text{LPML}=\overset{N}{\underset{i=1}{\sum }}\overset{J}{\underset{j=1}{\sum }}\text{log}\hat{\left(\text{CP}{\text{O}}_{ij}\right)}. \end{array}$$

The model with a larger LPML has a better fit to the data.

## 5. Simulation Studies

### 5.1. Simulation Study 1

This simulation study is conducted to evaluate the recovery performance of the Gibbs-slice sampling algorithm based on different simulation conditions.

#### 5.1.1. Simulation Design

The following manipulated conditions are considered: (a) test length *J* = 20 or 40 and (b) number of examinees *N* = 500, 1, 000, or 2, 000. Fully crossing different levels of these two factors yield six conditions (two test lengths × three sample sizes). Next, the true values of the parameters are given. True values of the item discrimination parameters *a*_*j*_ are generated from a uniform distribution, i.e., *a*_*j*_ ~ *U*(0.5, 2.5), *j* = 1, 2, …, *J*. The item difficulty parameters *b*_*j*_ are generated from a standardized normal distribution. The item guessing and slipping parameters (*c*_*j*_, γ_*j*_) are generated from *c*_*j*_ ~ *U*(0, 0.25) and γ_*j*_ ~ *U*(0, 0.25)I(γ_*j*_ < 1 − *c*_*j*_). The ability parameters of examinees θ_*i*_ are also generated from a standardized normal distribution. In addition, we adopt non-informative prior distributions for the item parameters, i.e., $a_{j}\sim N\left(0,10^{5}\right)\text{I}\left(0,+\infty \right)$, $b_{j}\sim N\left(0,10^{5}\right)$, *g*_*j*_ ~ Beta(1, 1), and γ_*j*_ ~ Beta(1, 1), *j* = 1, 2, …, *J*. The prior for the ability parameters is assumed to follow a standardized normal distribution owing to the model identification restrictions. One hundred replications are considered for each simulation condition.

#### 5.1.2. Convergence Diagnostics

To evaluate the convergence of parameter estimation, we only consider convergence in the case of minimum sample sizes owing to space limitations. That is, the test length is fixed at 20, and the number of examinees is 500. Two methods are used to check the convergence of our algorithm: the “eyeball” method to monitor convergence by visually inspecting the history plots of the generated sequences (Zhang et al., 2007), and the Gelman–Rubin method (Gelman and Rubin, 1992; Brooks and Gelman, 1998) to check the convergence of the parameters.

**Figure 1:**
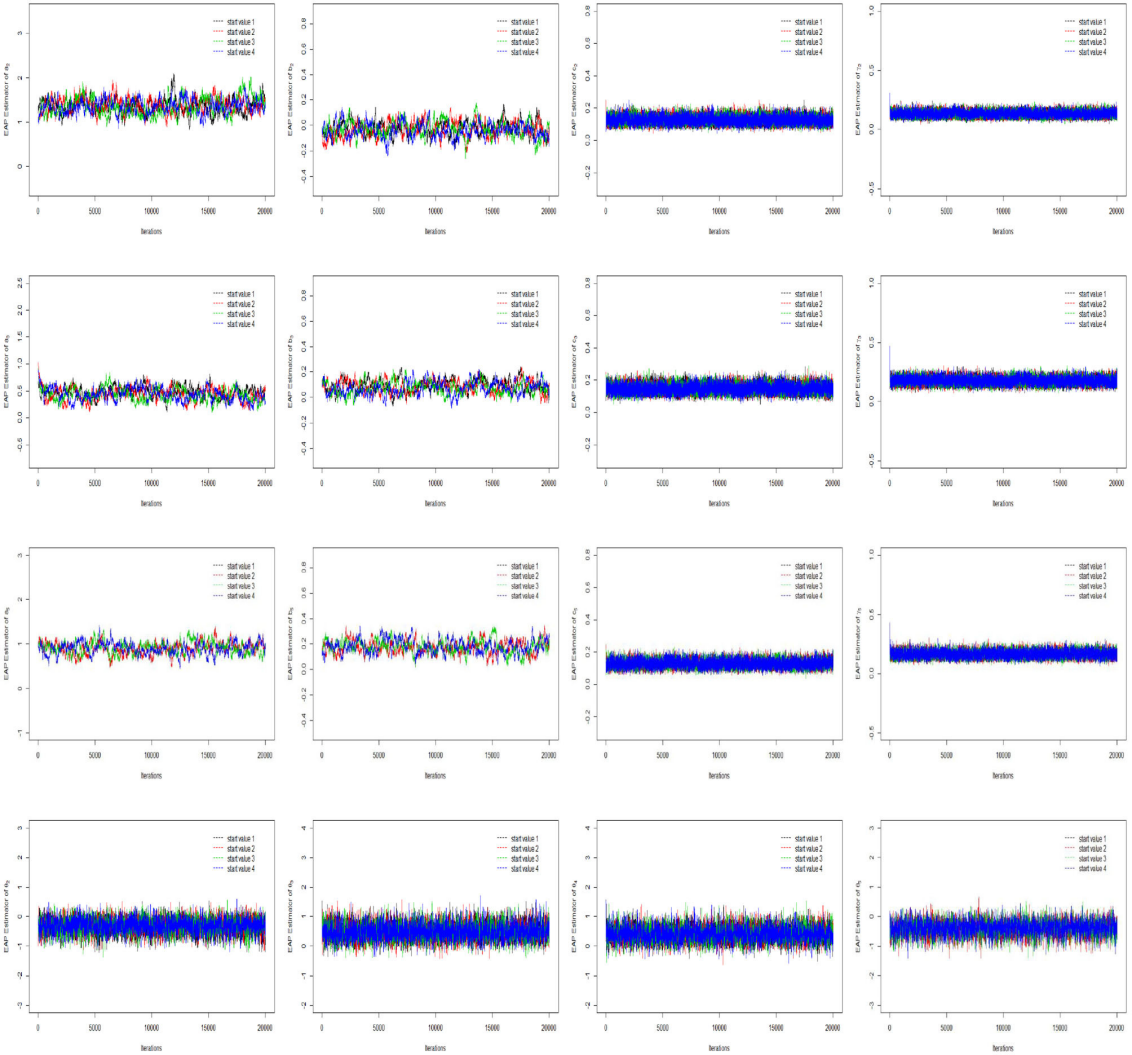
The trace plots of three randomly selected items and persons for the Simulation Study 1.

**Figure 2:**
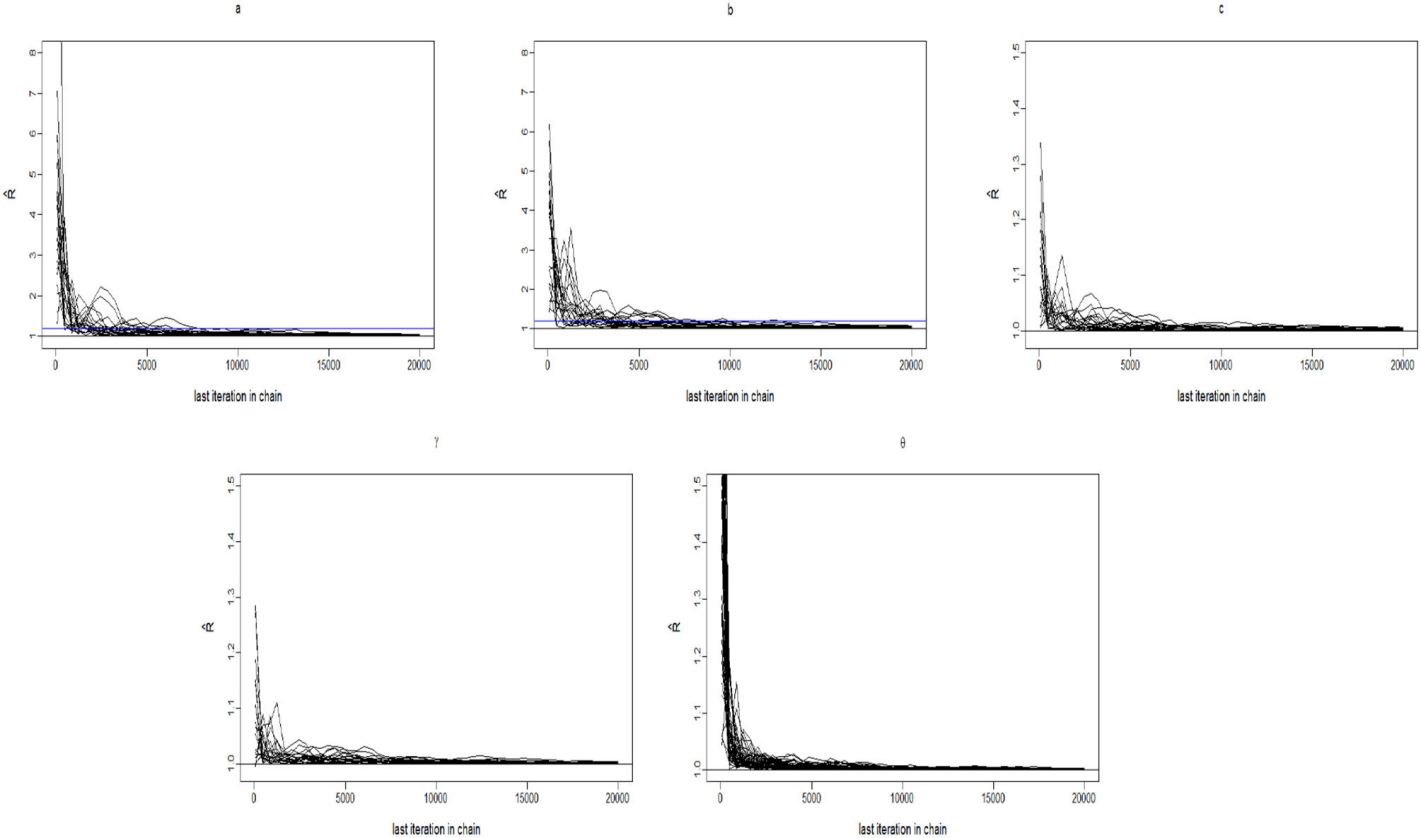
The trace plots of $\hat{R}$ for the Simulation Study 1.

The convergence of the Gibbs-slice sampling algorithm is checked by monitoring the trace plots of the parameters for consecutive sequences of 20,000 iterations. The first 10,000 iterations are set as the burn-in period. As an illustration, four chains started at overdispersed starting values are run for each replication. The trace plots of three randomly selected items and persons are shown in Figure 1. In addition, the potential scale reduction factor (PSRF) ($\hat{R}$; Brooks and Gelman, 1998) values of all item and person parameters are shown in Figure 2. We find that the PSRF values of all parameters are <1.2, which ensures that all chains converge as expected.

#### 5.1.3. Item Parameter Recovery

The accuracy of the parameter estimates is measured by four evaluation criteria, namely, the Bias, mean squared error (MSE), standard deviation (SD), and coverage probability (CP) of the 95% highest probability density interval (HPDI) statistics. Let η be the parameter of interest. Assume that *M* = 100 data sets are generated. Also, let ${\hat{\eta }}^{\left(m\right)}$ and SD^(*m*)^(η) denote the posterior mean and the posterior standard deviation of η obtained from the *m*th simulated data set for *m* = 1, …, *M*. The Bias for the parameter η is defined as

(24) $$\begin{array}{l} \text{Bias}\left(\eta \right)=\frac{1}{M}\overset{M}{\underset{m=1}{\sum }}\left({\hat{\eta }}^{\left(m\right)}-\eta \right), \end{array}$$

the MSE for η is defined as

(25) $$\begin{array}{l} \text{MSE}\left(\eta \right)=\frac{1}{M}\overset{M}{\underset{m=1}{\sum }}{\left({\hat{\eta }}^{\left(m\right)}-\eta \right)}^{2}, \end{array}$$

and the average of the posterior standard deviation is defined as

(26) $$\begin{array}{l} \text{SD}\left(\eta \right)=\frac{1}{M}\overset{M}{\underset{m=1}{\sum }}\text{S}{\text{D}}^{\left(m\right)}\left(\eta \right). \end{array}$$

Bias and MSE are important criteria used to evaluate the accuracy of parameter estimation in a simulation study. These criteria are used to investigate the relative distance between the parameter estimator and the true value. The greater the distance between the parameter estimator and the true value, the lower is the accuracy of parameter estimation and the poorer is the performance of the algorithm. However, for real data analysis, it is impossible to calculate Bias and MSE. The SD, on the other hand, can be calculated from the posterior samples of a Markov chain in simulation studies and real data analysis. In our simulation study, we calculate the average SD through repeated experiments to eliminate the error caused by randomness in a single simulation experiment.

The coverage probability is defined as

(27) $$\begin{array}{l} \text{CP}\left(\eta \right)=\frac{\#\text{ of }95\%\text{ HPDIs containing }\eta \text{ in }M\text{ simulated data sets}}{M}. \end{array}$$

The average Bias, MSE, SD, and CP for item parameters based on six different simulation conditions are shown in Table 1. The following conclusions can be drawn.

1. Given the total test length, when the number of individuals increases from 500 to 2,000, the average MSE and SD for discrimination, difficulty guessing, and slipping parameters show a decreasing trend. For example, let us consider a total test length of 20 items. When the number of examinees increases from 500 to 2,000, the average MSE and the average SD of all discrimination parameters decrease from 0.0625 to 0.0474 and from 0.1460 to 0.0759, respectively. The average MSE and the average SD of all difficulty parameters decrease from 0.0505 to 0.0263 and from 0.0559 to 0.0260, respectively. The average MSE and the average SD of all guessing parameters decrease from 0.0092 to 0.0023 and from 0.0247 to 0.0156, respectively. The average MSE and the average SD of all slipping parameters decrease from 0.0060 to 0.0025 and from 0.0260 to 0.0166, respectively.
2. Under the six simulated conditions, the average CPs of the discrimination, difficulty guessing, and slipping parameters are about 0.9500.
3. When the number of examinees is fixed at 500, 1,000, or 2,000, and the number of items is fixed at 40, the average MSE and SD show that the recovery results of the discrimination, difficulty, guessing, and slipping parameters are close to those in the case where the total test length is 20, which indicates that the Gibbs-slice sampling algorithm is stable and there is no reduction in accuracy owing to an increase in the number of items.

**Table 1:** Evaluating the accuracy of item parameters based on six different simulated conditions in Simulation Study 1.

	**No. of examinees 500**				**No. of examinees 1,000**				**No. of examinees 2,000**			
**Item parameter**	**Bias**	**MSE**	**SD**	**CP**	**Bias**	**MSE**	**SD**	**CP**	**Bias**	**MSE**	**SD**	**CP**
**NO. OF ITEMS = 20**												
Discrimination^a^	−0.0087	0.0625	0.1460	0.9514	−0.0217	0.0513	0.1037	0.9504	0.0005	0.0474	0.0759	0.9486
Difficulty^b^	−0.0000	0.0505	0.0559	0.9385	0.0000	0.0389	0.0390	0.9412	−0.0000	0.0263	0.0260	0.9285
Guessing^c^	−0.0215	0.0092	0.0247	0.9325	−0.0453	0.0045	0.0193	0.9378	−0.0830	0.0023	0.0156	0.9515
Slipping^γ^	0.0132	0.0060	0.0260	0.9342	−0.0176	0.0038	0.0217	0.9628	−0.0558	0.0025	0.0166	0.9548
**NO. OF ITEMS = 40**												
Discrimination^a^	−0.0029	0.0842	0.1482	0.9546	−0.0035	0.0705	0.0962	0.9390	−0.0129	0.0594	0.0638	0.9781
Difficulty^b^	−0.0000	0.0443	0.0561	0.9543	−0.0000	0.0325	0.0389	0.9495	0.0000	0.0224	0.0267	0.9652
Guessing^c^	−0.0238	0.0075	0.0250	0.9385	−0.0625	0.0059	0.0201	0.9322	−0.0677	0.0033	0.0154	0.9418
Slipping^γ^	−0.0061	0.0035	0.0264	0.9310	−0.0169	0.0025	0.0209	0.9438	−0.0407	0.0024	0.0152	0.9422

*Note that the Bias, MSE, SD, and CP denote the average Bias, MSE, SD, and CP for the parameters*.

a *Discrimination parameters*,

b *Difficulty parameters*,

c *Guessing parameters*,

γ *Slipping parameters*.

In summary, the Gibbs-slice sampling algorithm provides accurate estimates of the item parameters in term of various numbers of examinees and items. Next, we will explain why the Bias criterion is useful, and why it seems irrelevant in the simulation study.

If we want to determine whether our algorithm estimates the parameter accurately, we need more information to infer the parameter, which requires a large sample size. Here, Bias is an important criterion to evaluate the accuracy of parameter estimation. Let us give an example to illustrate the role of Bias. In Simulation Study 1, suppose that we investigate the accuracy of the algorithm in estimating a discrimination parameter. When the number of examinees increases from 500 to 2,000, the Bias of the discrimination parameter should show a decreasing trend. The result of Bias reduction further verifies that a greater number of samples are needed to improve the accuracy of parameter estimation.

In Simulation Study 1, we cannot enumerate the Bias of each item parameter one by one because there are too many simulation conditions and we are subject to space limitations. Therefore, we choose to calculate the average Bias of the parameter of interest. Next, we take the discrimination parameters as an example to further explain why Bias seems irrelevant in Simulation Study 1. Suppose that we have obtained 40 Biases of discrimination parameters, that the Bias values of these 40 discrimination parameters are either positive or negative, and that the average Bias of all 40 items is close to 0. However, the near-zero value of the average Bias does not show whether the parameter estimation is accurate or the result is caused by the positive and negative superposition of the 40 Biases. In fact, we find that the Bias for each item discrimination parameter show a decreasing trend with increasing number of examinees. To sum up, we do not analyze the results of the average Bias in the simulation studies, but Bias is indeed an important criterion to evaluate the accuracy of each parameter estimation.

#### 5.1.4. Ability Parameter Recovery

Next, we evaluate the recovery of the latent ability using four accuracy evaluation criteria. The following conclusions can be obtained from Table 2.

1. Given a fixed number of examinees (500, 1,000, or 2,000), when the number of items increases from 20 to 40, the average MSE and SD for the ability parameters also show a decreasing trend.
2. Under the six simulated conditions, the average CP of the ability is also about 0.9500.
3. Given a fixed number of examinees (500, 1,000, or 2,000), when the number of items increases from 20 to 40, the correlation between the estimates and the true values tends to increase. For example, for 500 examinees, when the number of items increases from 20 to 40, the correlation between the estimates and the true values increases from 0.8631 to 0.9102.
4. Given a fixed number of items (20 or 40), when the number of examinees increases from 500 to 2,000, the correlation between the estimates and the true values remains basically the same.

**Table 2:** Evaluating the accuracy of person parameters based on six different simulated conditions in Simulation Study 1.

**No. of**	**No. of**	**Bias**	**MSE**	**SD**	**CP**	**Correlation with**
**items**	**examinees**					**true value**
20	500	0.0545	0.2783	0.2523	0.9428	0.8631
	1,000	0.0149	0.2923	0.2636	0.9675	0.8764
	2,000	0.0052	0.3341	0.2961	0.9322	0.8599
40	500	0.0315	0.2346	0.2180	0.9274	0.9102
	1,000	0.0764	0.2553	0.2343	0.9626	0.9182
	2,000	0.0439	0.3042	0.2866	0.9542	0.9225

*Note that the Bias, MSE, SD, and CP denote the average Bias, MSE, SD, and CP for the ability parameters*.

In summary, it is shown again that the Gibbs-slice sampling algorithm is effective and that the estimated results are accurate under various simulation conditions.

### 5.2. Simulation Study 2

Culpepper (2016) conducted an additional simulation study to confirm that the guessing and slipping parameters could give good recovery results in the process of Gibbs sampling regardless of whether informative or non-informative priors were used. Therefore, in this simulation study, we also adopt non-informative prior distributions for the guessing and slipping parameters in the Gibbs step to eliminate biased estimation of parameters due to wrong choices of the prior distributions, i.e., *c* ~ Beta(1, 1) and γ ~ Beta(1, 1), and we focus on the influence of different prior distributions on the accuracy of parameter estimation in the process of implementing the slice sampling algorithm. Note that in this simulation study, we do not focus on the accuracy of the guessing and slipping parameters, since Culpepper (2016) has already verified the accuracy of these two parameters in the case of the Gibbs algorithm under different types of prior distributions.

This simulation study is designed to show that the slice sampling algorithm is sufficiently flexible to recover various prior distributions of the item (discrimination and difficulty) and person parameters, and to address the sensitivity of the algorithm with different priors. Three types of prior distributions are examined: informative priors, non-informative priors, and inappropriate priors.

#### 5.2.1. Simulation Design

The number of the examinees *N* = 1, 000, and the test length *J* = 20. The true values for the items and persons are the same as in Simulation Study 1. One hundred replications are considered for each simulation condition. The following three kinds of prior distributions are considered in implementing the slice sampling algorithm:

- informative prior: *a* ~ log*N*(0, 1), *b* ~ *N*(0, 1), and θ ~ *N*(0, 1);
- non-informative prior: *a* ~ *N*(0, 1000)I(0, +∞), *b* ~ Uniform(−1000, 1000), and θ ~ *N*(0, 1000);
- inappropriate prior: (1) *a* ~ Exp(1), *b* ~ *t*(1), and θ ~ *t*(1); (2) *a* ~ Gamma(3, 2), *b* ~ Cauchy(1, 3), and θ ~ Cauchy(1, 3).

**Figure 3:**
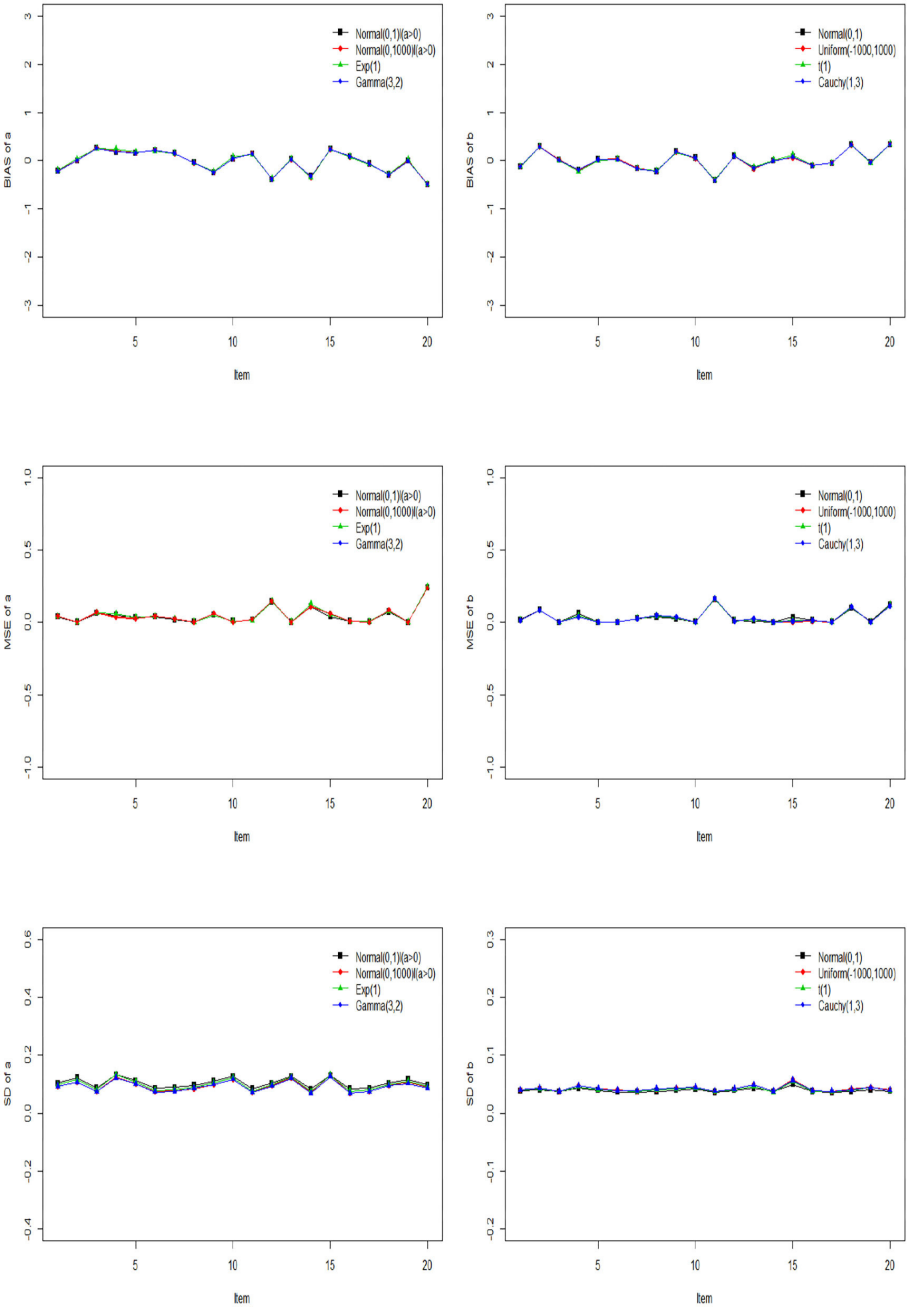
The Bias, MSE, and SD of discrimination and difficulty parameters based on different priors.

The Gibbs-slice sampling algorithm is iterated 20,000 times. The first 10,000 iterations are discarded as burn-in. The PSRF values of all parameters are <1.2. The Bias, MSE, and SD of ***a*** and ***b*** based on the three kinds of prior distribution are shown in Figure 3.

#### 5.2.2. Item Parameter Recovery

From Figure 3, we can see that the Bias, MSE, and SD of ***a*** and ***b*** are almost the same under different prior distributions. This shows that accuracy of parameter estimation can be guaranteed by the slice sampling algorithm, no matter what prior distribution is chosen, as long as the values sampled from this distribution belong to a reasonable parameter support set. In addition, the Bias, MSE and SD of ***a*** and ***b*** fluctuate around 0, which shows that the slice sampling algorithm is accurate and effective in estimating the item parameters.

#### 5.2.3. Ability Parameter Recovery

Next, we evaluate the recovery of the latent ability based on different prior distributions in Table 3. We find that the MSE of ability parameters is between 0.2676 and 0.3014, and the corresponding SD is between 0.2436 and 0.3026 for all three kinds of prior distribution, which indicates that the choice of prior distribution has little impact on the accuracy of the ability parameters. In summary, the slice sampling algorithm is accurate and effective in estimating the person parameters. It is not sensitive to the specification of priors.

**Table 3:** Evaluating the accuracy of person parameters based on different prior distributions in Simulation Study 2.

**Parameter**	**Accuracy evaluation index**	**Prior distribution**						
		***N*(0, 1)**		***N*(0, 1, 000)**		***t*(1)**		***Cauchy*(1, 3)**
θ	Bias	0.0064		0.0149		0.0087		0.0238
	MSE	0.2676		0.2923		0.3014		0.2713
	SD	0.2436		0.3026		0.2810		0.2983

*Note that the Bias, MSE, and SD denote the average Bias, MSE, and SD for the ability parameters*.

### 5.3. Simulation Study 3

In this simulation study, we use two Bayesian model assessment criteria to evaluate the model fittings. Two issues warrant further study. The first is whether the two criteria can accurately identify the true models under different design conditions. The second is that we study the phenomena of over-fitting and under-fitting between the true model and the fitting models.

#### 5.3.1. Simulation Design

In this simulation, a number of individuals *N* = 1, 000 is considered and the test length is fixed at 40. Three item response models are considered: the 2PL, 3PL, and 4PL models. Thus, we evaluate model fitting in the following three cases:

- Case 1: 2PL model (true model) vs. 2PL model, 3PL model, or 4PL model (fitted model).
- Case 2: 3PL model (true model) vs. 2PL model, 3PL model, or 4PL model (fitted model).
- Case 3: 4PL model (true model) vs. 2PL model, 3PL model, or 4PL model (fitted model).

The true values and prior distributions for the parameters are specified in the same way as in Simulation Study 1. To implement the MCMC sampling algorithm, chains of length 20,000 with an initial burn-in period of 10,000 are chosen. There are 100 replications for each simulation condition. The potential scale reduction factor (PSRF; Brooks and Gelman, 1998) values of all item and person parameters for each simulation condition are <1.2. The results of Bayesian model assessment based on the 100 replications are shown in Table 4.

**Table 4:** The results of Bayesian model assessment in the Simulation Study 3.

	**True model**			**2PL**	**3PL**	**4PL**
Fitted model	2PL	DIC	*Q*_1_	**29319.0702**	30539.6070	34676.5622
			Median	**29333.1917**	30567.3468	34702.6678
			*Q*_3_	**29341.0284**	30591.9937	34722.2367
			IQR	21.9582	52.3867	45.6745
		LPML	*Q*_1_	−**14888.3688**	−15543.2057	−17701.0943
			Median	−**14881.2617**	−15532.2437	−17686.7704
			*Q*_3_	−**14875.4347**	−15515.0444	−17670.9324
			IQR	12.9341	28.1613	30.1319
	3PL	DIC	*Q*_1_	32431.0873	**24857.3160**	32648.0788
			Median	32436.0587	**24866.9338**	32653.6306
			*Q*_3_	32442.8955	**24878.2528**	32660.3940
			IQR	11.8082	20.9368	12.3152
		LPML	*Q*_1_	−16413.9390	−**12528.9444**	− 16462.3200
			Median	−16410.4056	−**12523.6985**	− 16458.5988
			*Q*_3_	−16406.8835	−**12517.8991**	− 16453.9725
			IQR	7.0555	11.0453	8.3427
	4PL	DIC	*Q*_1_	35560.7897	28870.1192	**27768.0166**
			Median	35583.7535	28880.2811	**27775.8655**
			*Q*_3_	35611.7761	28890.8003	**27780.0024**
			IQR	50.9863	20.6810	11.9857
		LPML	*Q*_1_	−18320.2375	−14603.6126	−**13965.3888**
			Median	−18302.6986	−14597.3004	−**13963.2251**
			*Q*_3_	−18288.7386	−14593.5979	−**13958.0409**
			IQR	31.4988	10.0147	7.3479

*Note that the boldface values indicate that the corresponding model is the best fitted model with the smallest DIC and largest LPML values*.

From Table 4, we find that when the 2PL model is the true model, the 2PL model is chosen as the best-fitting model according to the results of DIC and LPML, which is what we expect to see. The medians of DIC and LPML are, respectively 29,333.1917 and −14,881.2617. The second best-fitting model is the 3PL model. The differences between the 2PL and 3PL models in the medians of DIC and LPML are −1234.1551 and 650.9820, respectively. The 4PL model is the worst model to fit the data. This is because the data are generated from a simple 2PL model, and the complex 4PL model is used to fit this data, which leads to over-fitting. The differences between the 2PL and 4PL models in the medians of DIC and LPML are −5369.4761 and 2805.5087, respectively. When the 3PL model is the true model, the DIC and LPML consistently choose the 3PL model as the best-fitting model, with the corresponding median values being 24,866.9338 and −12,523.6985, respectively. The second best-fitting model is the 2PL model. The differences between the 3PL and 4PL models in the medians of DIC and LPML are −7786.6968 and 3934.9003, respectively, while the corresponding differences between the 3PL and 2PL models are −7569.1249 and 3886.7071. This shows that when the data are generated from the 3PL model, the simple 2PL model is more appropriate to fit the data compared with the complex 4PL model. When the 4PL model is the true model, the two criteria consistently select the 4PL model as the best-fitting model. The other two models suffer from serious under-fitting. The differences between the 4PL and 2PL models in the medians of DIC and LPML are −7807.8880 and 4339.4735, respectively, while the corresponding differences between the 4PL and 3PL models are −1104.4156 and 634.0753. The failure to select the 2PL (3PL) model is attributed to the under-fitting caused by a few parameters. That is, the guessing and slipping parameters in the 4PL model play an important role in adjusting the probability of the tail of the item characteristic curve. In summary, the Bayesian assessment criteria are effective for identifying the true models and can be used in the following empirical example.

## 6. Empirical Example

**Figure 4:**
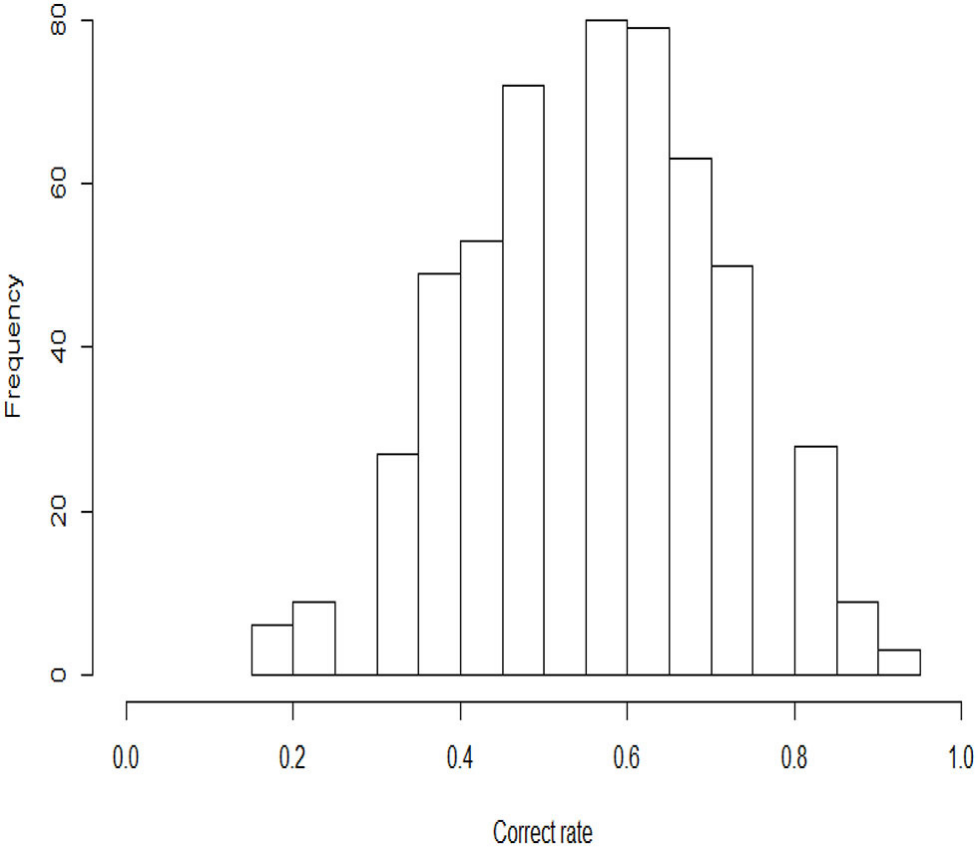
Frequency histograms of the correct rates for 548 examinees.

In this example, the 2015 computer-based PISA (Program for International Student Assessment) science data are used. Among the many countries that have participated in this computer-based assessment of sciences, we choose students from the USA as the object of analysis. The original sample size of students is 658, and 110 students with Not Reached (original code 6) or Not Response (original code 9) are removed, with Not Reached and Not Response (omitted) being treated as missing data. The final 548 students answer 16 items. All 16 items are scored using a dichotomous scale. The descriptive statistics for these PISA data are shown in Table 5. We find that three items, DR442Q05C, DR442Q06C, and CR442Q07S, have lower correct rates than the other items, with the corresponding values being 25.7, 23.2, and 28.5%, respectively. The correct rate represents the proportion at which all examinees answer each item correctly. Moreover, the four items with the highest correct rates are CR101Q02S (87.6%), CR083Q02S (83.6%) DR442Q02C (80.1%), and CR101Q04S (80.1%). The frequency histogram of the correct rates for the 548 examinees is shown in Figure 4.

**Table 5:** The descriptive statistics for PISA 2015 released computer-based sciences items.

**Item**	**Item code**	**Correct rate (%)**	**Item**	**Item code**	**Correct rate (%)**
1	CR083Q01S	54.2	9	CR442Q07S	28.5
2	CR083Q02S	83.6	10	CR245Q01S	53.8
3	CR083Q03S	75.2	11	CR245Q02S	60.0
4	CR083Q04S	66.6	12	CR101Q01S	43.6
5	DR442Q02C	80.1	13	CR101Q02S	87.6
6	DR442Q03C	76.5	14	CR101Q03S	57.7
7	DR442Q05C	25.7	15	CR101Q04S	80.1
8	DR442Q06C	23.1	16	CR101Q05S	48.7

*Note that the correct rate represents the percentage of all examinees who correctly answer each item*.

### 6.1. Bayesian Model Assessment

We consider three models to fit the PISA data: the 2PL, 3PL, and 4PL models. In the estimation procedure, the same non-informative priors as in Simulation Study 1 are utilized for the unknown parameters. In all of the Bayesian computations, we use 20,000 MCMC samples after a burn-in of 10,000 iterations for each model to compute all posterior estimates. The convergence of the chains is checked by PSRF. The PSRF values of all item and ability parameters for each model are <1.2. On this basis, the results of Bayesian model assessment for the PISA data are shown in Table 6.

**Table 6:** The results of Bayesian model assessment for the PISA data.

**Model**	**DIC**	**LPML**
2PL model	14206.9508	−7290.7545
3PL model	12230.3819	−6168.9428
4PL model	**10854.2075**	− **5494.4088**

*Note that the boldface values indicate that the corresponding model is the best fitted model with the smallest DIC and largest LPML values*.

According to DIC and LPML in Table 6, we find that the 4PL model is the best-fitting model compared with the 2PL and 3PL models. The values of DIC and LPML are 10,854.2075 and −5494.4088, respectively. The second best-fitting model is the 3PL model. The differences between the 4PL and 3PL models in DIC and LPML are −1376.1744 and 674.5340, respectively. This shows that the introduction of slipping parameters in the 3PL model is sufficient to fit these PISA data. The worst-fitting model is the 2PL model. This is attributed to the relatively simple structure of this model, which makes it unable to describe changes in probability at the end of the item characteristic curve caused by guessing or slipping. The differences between the 4PL and 2PL models in DIC and LPML are −3353.7433 and 1796.3457, respectively.

Next, we will use the 4PL model to analyze the PISA data in detail based on the results of the model assessment.

### 6.2. Analysis of Item Parameters

The estimated results for the item parameters are shown in Table 7, from which we find that the expected a posteriori (EAP) estimations of the 11 item discrimination parameters are greater than one. This indicates that these items can distinguish the differences between abilities well. The five items with the lowest discrimination are items 16 (CR101Q05S), 10 (CR245Q01S), 12 (CR101Q01S), 2 (CR083Q02S), and 5 (DR442Q02C) in turn. The EAP estimates of the discrimination parameters for these five items are 0.6681, 0.6792, 0.7348, 0.8083, and 0.8901. In addition, the EAP estimates of seven of the difficulty parameters are less than zero, which indicates that these seven items are easier than the other nine items. The five most difficult items are items 8 (DR442Q06C), 7 (DR442Q05C), 9 (CR442Q07S), 12 (CR101Q01S), and 16 (CR101Q05S) in turn. The EAP estimates of the difficulty parameters for these five items are 1.2528, 1.2203, 1.0804, 0.4521, and 0.3102. The corresponding correct rates in Table 5 for these five items are 23.1, 25.7, 28.5, 43.63 and 48.7%, respectively. The most difficult five items have low correct rates, which is consistent with our intuition. The EAP estimates of the guessing parameters for the 16 items range from 0.0737 to 0.1840. The five items with the highest guessing parameters are items 2 (CR083Q02S), 5 (DR442Q02C), 13 (CR101Q02S), 15 (CR101Q04S), and 3 (CR083Q03S) in turn. The EAP estimates of the guessing parameters for these five items are 0.1840, 0.1791, 0.1790, 0.1673, and 0.3102. We find that the five items with high guessing parameters also have high correct rates. The corresponding correct rates for these five items are 83.6, 80.1, 87.6, 80.1, and 75.2%. This shows that these five items are more likely to be guessed correctly than the other 11 items. In addition, the five easiest slipping items are items 8 (DR442Q06C), 7 (DR442Q05C), 9 (CR442Q07S), 12 (CR101Q01S), and 16 (CR101Q05S) in turn. The EAP estimates of the slipping parameters for these five items are 1.785, 1.619, 1.581, 0.1481, and 0.1431. We find that the more difficult an item is, the more likely is the examinee to slip in answering it, which leads to a reduction in the correct rate. The SDs of the discrimination parameters range from 0.0897 to 0.1719, those of the difficulty parameters from 0.0512 to 0.0966, those of the guessing parameters from 0.0147 to 0.0363, and those of the slipping parameters from 0.0118 to 0.0339.

**Table 7:** The estimation results of item parameter for the PISA data.

**PARM**	**EAP**	**SD**	**HPDI**	**PARM**	**EAP**	**SD**	**HPDI**
*a*_1_	1.0416	0.1227	[0.8215, 1.2856]	*b*_1_	0.1939	0.0615	[0.0861, 0.3222]
*a*_2_	0.8083	0.1316	[0.5715, 1.0665]	*b*_2_	−0.8496	0.0815	[−0.9936, −0.6793]
*a*_3_	1.1171	0.1513	[0.8327, 1.4101]	*b*_3_	−0.5071	0.0625	[−0.6214, −0.3699]
*a*_4_	1.1119	0.1308	[0.8813, 1.3996]	*b*_4_	−0.1947	0.0563	[−0.3030, −0.0876]
*a*_5_	0.8901	0.1034	[0.6847, 1.0933]	*b*_5_	−0.6969	0.0623	[−0.8230, −0.5741]
*a*_6_	1.2772	0.1719	[0.9642, 1.6355]	*b*_6_	−0.5966	0.0700	[−0.7525, −0.4675]
*a*_7_	1.3404	0.1348	[1.0800, 1.5839]	*b*_7_	1.2203	0.0778	[1.0635, 1.3738]
*a*_8_	1.1202	0.1608	[0.7827, 1.4713]	*b*_8_	1.2528	0.0966	[1.0313, 1.4246]
*a*_9_	1.2377	0.1475	[0.9338, 1.5149]	*b*_9_	1.0804	0.0819	[0.9117, 1.2155]
*a*_10_	0.6792	0.1125	[0.4780, 0.9079]	*b*_10_	0.1669	0.0640	[0.0423, 0.2832]
*a*_11_	1.0720	0.1214	[0.8432, 1.3184]	*b*_11_	0.0258	0.0512	[−0.0617, 0.1330]
*a*_12_	0.7348	0.0897	[0.5528, 0.9035]	*b*_12_	0.4521	0.0548	[0.3448, 0.5506]
*a*_13_	1.1994	0.1706	[0.8682, 1.5513]	*b*_13_	−1.1843	0.0841	[−1.3510, −1.0305]
*a*_14_	1.0083	0.1219	[0.7666, 1.2336]	*b*_14_	0.0985	0.0525	[0.0029, 0.2053]
*a*_15_	1.2047	0.1707	[0.8618, 1.5329]	*b*_15_	−0.7719	0.0667	[−0.9095, −0.6543]
*a*_16_	0.6681	0.0924	[0.4999, 0.8482]	*b*_16_	0.3102	0.0584	[0.2012, 0.4321]
*c*_1_	0.1344	0.0254	[0.0870, 0.1853]	γ_1_	0.1170	0.0225	[0.0738, 0.1616]
*c*_2_	0.1840	0.0363	[0.1137, 0.2545]	γ_2_	0.0736	0.0142	[0.0466, 0.1023]
*c*_3_	0.1650	0.0315	[0.1065, 0.2285]	γ_3_	0.0804	0.0155	[0.0506, 0.1106]
*c*_4_	0.1532	0.0292	[0.1006, 0.2137]	γ_4_	0.0950	0.0182	[0.0605, 0.1306]
*c*_5_	0.1791	0.0343	[0.1131, 0.2461]	γ_5_	0.0781	0.0149	[0.0495, 0.1077]
*c*_6_	0.1607	0.0309	[0.1014, 0.2210]	γ_6_	0.0749	0.0148	[0.0458, 0.1032]
*c*_7_	0.0737	0.0147	[0.0459, 0.1023]	γ_7_	0.1619	0.0314	[0.1034, 0.2261]
*c*_8_	0.0805	0.0152	[0.0507, 0.1096]	γ_8_	0.1785	0.0339	[0.1145, 0.2470]
*c*_9_	0.0842	0.0159	[0.0549, 0.1165]	γ_9_	0.1581	0.0307	[0.0983, 0.2178]
*c*_10_	0.1561	0.0279	[0.1024, 0.2115]	γ_10_	0.1313	0.0248	[0.0832, 0.1786]
*c*_11_	0.1485	0.0268	[0.0996, 0.2035]	γ_11_	0.1028	0.0197	[0.0646, 0.1408]
*c*_12_	0.1361	0.0243	[0.0897, 0.1842]	γ_12_	0.1481	0.0275	[0.0967, 0.2040]
*c*_13_	0.1790	0.0354	[0.1118, 0.2484]	γ_13_	0.0607	0.0118	[0.0368, 0.0827]
*c*_14_	0.1469	0.0268	[0.0952, 0.1991]	γ_14_	0.1100	0.0211	[0.0697, 0.1523]
*c*_15_	0.1673	0.0322	[0.1057, 0.2299]	γ_15_	0.0716	0.0143	[0.0444, 0.1006]
*c*_16_	0.1505	0.0266	[0.0991, 0.2028]	γ_16_	0.1431	0.0268	[0.0931, 0.1960]

*PARM denotes parameter, EAP is the expected a posteriori estimation, SD denotes the standard deviation, and HPDI denotes the highest probability density interval*.

### 6.3. Analysis of Person Parameters

**Figure 5:**
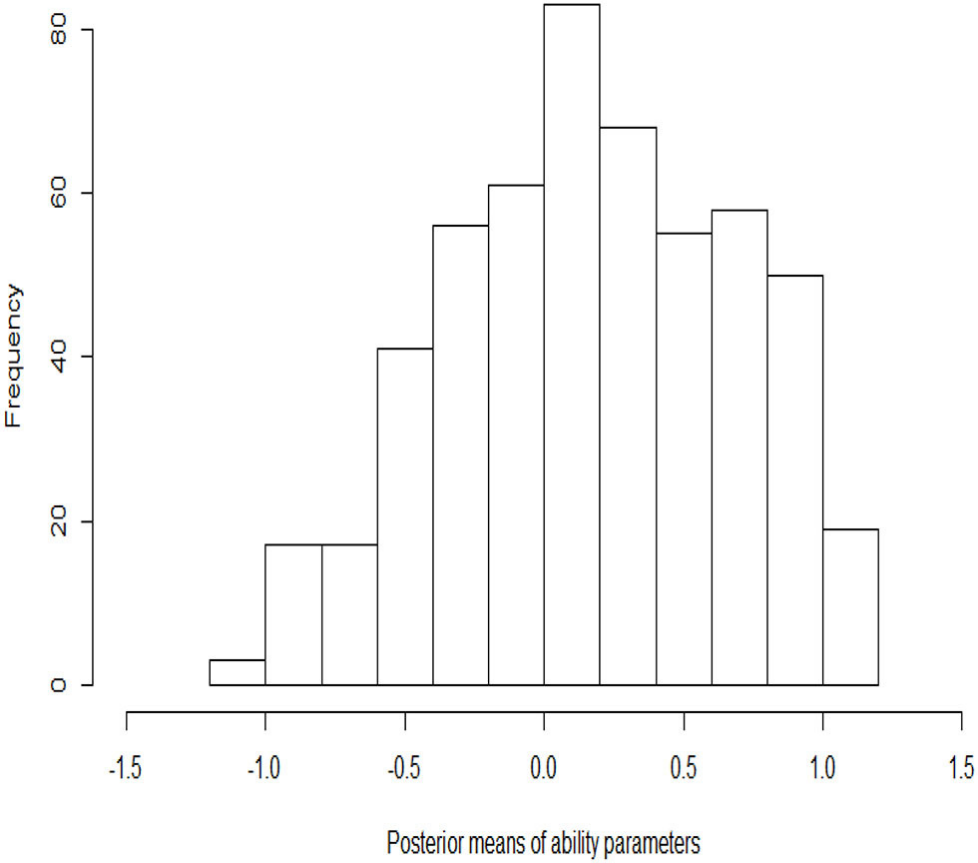
The histograms of the posterior estimates of ability parameters.

The histograms of the posterior estimates of the ability parameters are shown in Figure 5. Most of the estimated abilities of the examinees are near zero. The number of examinees with high ability (the estimates are between 0 and 1.2) is more than the number with low ability (the estimates are between −1.2 and 0). The ability parameter posterior histogram is consistent with the frequency histogram of the correct rate (Figure 4). That is, the trend of change in the correct rate in the histogram is same as that in the ability posterior histogram. The number of examinees with high correct rate is more than the number with low correct rate. It is once again verified that the results of the estimation are accurate.

## 7. Discussion

In this paper, an efficient Gibbs-slice sampling algorithm in a fully Bayesian framework has been proposed to estimate the 4PL model. This algorithm, as its name suggests, can be conceived of as an extension of the Gibbs algorithm. The sampling process consists of two parts. One part is the Gibbs algorithm, which is used to update the guessing and slipping parameters when non-informative uniform priors are employed for cases that are prototypical of educational and psychopathology items. This part implements sampling by using a conjugate prior and greatly increases efficiency. The other part is the slice sampling algorithm, which samples the 2PL IRT model from the truncated full conditional posterior distribution by introducing auxiliary variables. The motivations for the slice sampling algorithm are manifold. First, this algorithm has the advantage of flexibility in the choice of prior distribution to obtain samples from the full conditional posterior distributions, rather than being restricted to using the conjugate distributions as in the Gibbs sampling process, which is also limited to the normal ogive framework. This allows the use of informative priors, non-informative priors, and inappropriate priors for the item parameters. Second, the Metropolis–Hastings algorithm depends on the proposal distributions and variances (tuning parameters) and is sensitive to step size. If the step size is too small, the chain will take longer to traverse the target density. If the step size is too large, there will be inefficiencies due to a high rejection rate. However, the slice sampling algorithm can automatically tune the step size to match the local shape of the target density and draw samples with acceptance probability equal to one. Thus, it is easier and more efficient to implement.

However, the computational burden of the Gibbs-slice sampling algorithm becomes intensive, especially when a large numbers of examinees or items are considered, or a large MCMC sample size is used. Therefore, it is desirable to develop a standalone R package associated with C++ or Fortran software for more a extensive large-scale assessment program. In fact, the new algorithm based on auxiliary variables can be extended to estimate some more complex item response and response time models, for example, the graded response model or the Weibull response time model. Only DIC and LPML have been considered in this study, but other Bayesian model selection criteria such as marginal likelihoods may also be potentially useful to compare different IRT models. These extensions are beyond the scope of this paper but are currently under further investigation.

## Data Availability Statement

Publicly available datasets were analyzed in this study. This data can be found here: http://www.oecd.org/pisa/data/.

## Author Contributions

JZ completed the writing of the article and provided article revisions. JL provided original thoughts. JZ, JL, HD, and ZZ provided key technical support. All authors contributed to the article and approved the submitted version.

## Conflict of Interest

The authors declare that the research was conducted in the absence of any commercial or financial relationships that could be construed as a potential conflict of interest.

## References

[B1] Albert J. H. (1992). Bayesian estimation of normal ogive item response curves using Gibb ssampling. J. Educ. Stat. 17, 251–269.

[B2] Baker F. B. Kim S. H. (2004). Item Response Theory: Parameter Estimation Techniques. New York, NY: Marcel Dekker.

[B3] Barton M. A. Lord F. M. (1981). An Upper Asymptote for the Three-Parameter Logistic Item Response Model. Princeton, NJ: Educational Testing Service.

[B4] Béguin A. A. Glas C. A. W. (2001). MCMC estimation of multidimensional IRT models. Psychometrika 66, 541–561. 10.1007/BF02296195

[B5] Birnbaum A. (1957). Efficient Design and Use of Tests of a Mental Ability for Various Decision-Making Problems. Series Report No. 58-16. Randolph Air Force Base, TX: USAF School of Aviation Medicine.

[B6] Birnbaum A. (1968). Some latent trait models and their use in inferring an examinee's ability, in Statistical Theories of Mental Test Scores, eds Lord F. M. Novick M. R. (Reading, MA: MIT Press), 397–479.

[B7] Bishop C. M. (2006). Slice sampling, in Pattern Recognition and Machine Learning, eds Jordan M. Kleinberg J. Schölkopf B. (New York, NY: Springer), 523–558.

[B8] Bock R. D. Aitkin M. (1981). Marginal maximum likelihood estimation of item parameters: application of an EM algorithm. Psychometrika 46, 443–459. 10.1007/BF02293801

[B9] Brooks S. P. Gelman A. (1998). Alternative methods for monitoring convergence of iterative simulations. J. Comput. Graph. Stat. 7, 434–455. 10.1080/10618600.1998.10474787

[B10] Chalmers R. P. (2012). mirt: A multidimensional item response theory package for the Renvironment. J. Stat. Softw. 48, 1–29. 10.18637/jss.v048.i06

[B11] Chang H.-H. Ying Z. (2008). To weight or not to weight? Balancing influence of initial items in adaptive testing. Psychometrika 73, 441–450. 10.1007/s11336-007-9047-7

[B12] Chen M.-H. Shao Q.-M. Ibrahim J. G. (2000). Monte Carlo Methods in Bayesian Computation. New York, NY: Springer.

[B13] Chib S. Greenberg E. (1995). Understanding the Metropolis-Hastings algorithm. Am. Stat. 49, 327–335. 10.1080/00031305.1995.10476177

[B14] Culpepper S. A. (2016). Revisiting the 4-parameter item response model: Bayesian estimation and application. Psychometrika 81, 1142–1163. 10.1007/s11336-015-9477-6 26400070

[B15] Damien P. Wakefield J. Walker S. (1999). Gibbs sampling for Bayesian non-conjugate and hierarchical models by auxiliary variables. J. R. Stat. Soc. Ser. B 61, 331–344. 10.1111/1467-9868.00179

[B16] Embretson S. E. Reise S. P. (2000). Item Response Theory for Psychologists. Mahwah, NJ: Erlbaum.

[B17] Ferrando P. J. (1994). Fitting item response models to the EPI-A impulsivity subscale. Educ. Psychol. Measure. 54, 118–127. 10.1177/0013164494054001016

[B18] Fox J.-P. (2010). Bayesian Item Response Modeling: Theory and Applications. New York, NY: Springer.

[B19] Fox J. P. (2005). Multilevel IRT using dichotomous and polytomous items. Br. J. Math. Stat. Psychol. 58, 145–172. 10.1348/000711005X38951 15969844

[B20] Fox J. P. Glas C. A. W. (2001). Bayesian estimation of a multilevel IRT model using Gibbs sampling. Psychometrika 66, 271–288. 10.1007/BF02294839

[B21] Fraley R. C. Waller N. G. Brennan K. A. (2000). An item response theory analysis of self-report measures of adult attachment. J. Pers. Soc. Psychol. 78, 350–365. 10.1037/0022-3514.78.2.350 10707340

[B22] Geisser S. Eddy W. F. (1979). A predictive approach to model selection. J. Am. Stat. Assoc. 74, 153–160. 10.1080/01621459.1979.10481632

[B23] Gelfand A. E. Dey D. K. Chang H. (1992). Model determination using predictive distributions with implementation via sampling-based methods (with discussion), in Bayesian Statistics, Vol. 4, eds Bernardo J. M. Berger J. O. Dawid A. P. Smith A. F. M. (Oxford: Oxford University Press), 147–167.

[B24] Gelfand A. E. Smith A. F. M. (1990). Sampling-based approaches to calculating marginal densities. J. Am. Stat. Assoc. 85, 398–409. 10.1080/01621459.1990.10476213

[B25] Gelman A. Rubin D. B. (1992). Inference from iterative simulation using multiple sequences. Stat. Sci. 7, 457–472. 10.1214/ss/1177011136

[B26] Geman S. Geman D. (1984). Stochastic relaxation, Gibbs distributions, and the Bayesian restoration of images. IEEE Trans. Pattern Anal. Machine Intell. 6, 721–741. 10.1109/TPAMI.1984.4767596 22499653

[B27] Gray-Little B. Williams V. S. L. Hancock T. D. (1997). An item response theory analysis of the Rosenberg Self-Esteem Scale. Pers. Soc. Psychol. Bull. 23, 443–451. 10.1177/0146167297235001

[B28] Green B. F. (2011). A comment on early student blunders on computer-based adaptive tests. Appl. Psychol. Measure. 35, 165–174. 10.1177/0146621610377080

[B29] Hastings W. K. (1970). Monte Carlo sampling methods using Markov chains and their applications. Biometrika 57, 97–109. 10.1093/biomet/57.1.97

[B30] Hessen D. J. (2005). Constant latent odds- ratios models and the Mantel-Haenszel null hypothesis. Psychometrika 70, 497–516. 10.1007/s11336-002-1040-6 20046835 PMC2798042

[B31] Hockemeyer C. (2002). A comparison of non-deterministic procedures for the adaptive assessment of knowledge. Psychol. Beiträge 44, 495–503.

[B32] Ibrahim J. G. Chen M.-H. Sinha D. (2001). Bayesian Survival Analysis. New York, NY: Springer.

[B33] Junker B. W. Sijtsma K. (2001). Cognitive assessment models with few assumptions, and connections with nonparametric item response theory. Appl. Psychol. Measure. 25, 258–272. 10.1177/01466210122032064

[B34] Lanza S. T. Foster M. Taylor T. K. Burns L. (2005). Assessing the impact of measurement specificity in a behavior problems checklist: An IRT analysis. Technical Report 05-75. The Pennsylvania State University; The Methodology Center, University Park, PA.

[B35] Liao W.-W. Ho R.-G. Yen Y.-C. Cheng H.-C. (2012). The four-parameter logistic item response theory model as a robust method of estimating ability despite aberrant responses. Soc. Behav. Pers. 40, 1679–1694. 10.2224/sbp.2012.40.10.1679

[B36] Loken E. Rulison K. (2010). Estimation of a four-parameter item response theory model. Br. J. Math. Stat. Psychol. 63, 509–525. 10.1348/000711009X474502 20030965

[B37] Lord F. M. Novick M. R. (1968). Statistical Theories of Mental Test Scores. Reading, MA: Addison-Wesley.

[B38] Lu J. Zhang J. W. Tao J. (2018). Slice-Gibbs sampling algorithm for estimating the parameters of a multilevel item response model. J. Math. Psychol. 82, 12–25. 10.1016/j.jmp.2017.10.005

[B39] Magis D. (2013). A note on the item information function of the four-parameter logistic model. Appl. Psychol. Measure. 37, 304–315. 10.1177/0146621613475471

[B40] Metropolis N. Rosenbluth A. W. Rosenbluth M. N. Teller A. H. Teller E. (1953). Equation of state calculations by fast computing machines. J. Chem. Phys. 21, 1087–1092. 10.1063/1.1699114 41342507

[B41] Neal R. (2003). Slice sampling. Ann. Stat. 31, 705–767. 10.1214/aos/1056562461

[B42] Ogasawara H. (2012). Asymptotic expansions for the ability estimator in item response theory. Comput. Stat. 27, 661–683. 10.1007/s00180-011-0282-0

[B43] Osgood D. W. McMorris B. J. Potenza M. T. (2002). Analyzing multiple-item measures of crime and deviance I: item response theory scaling. J. Quant. Criminol. 18, 267–296. 10.1023/A:1016008004010

[B44] Patz R. J. Junker B. W. (1999). A straight forward approach to Markov chain Monte Carlo methods for item response models. J. Educ. Behav. Stat. 24, 146–178. 10.3102/10769986024002146

[B45] Rasch G. (1960). Probabilistic Models for Some Intelligence and Attainment Tests. Copenhagen: Danish Institute for Educational Research.

[B46] Reise S. P. Waller N. G. (2003). How many IRT parameters does it take to model psychopathology items? Psychol. Methods 8, 164–184. 10.1037/1082-989X.8.2.164 12924813

[B47] Rouse S. V. Finger M. S. Butcher J. N. (1999). Advances in clinical personality measurement: an item response theory analysis of the MMPI-2 PSY-5 scales. J. Pers. Assess. 72, 282–307. 10.1207/S15327752JP720212

[B48] Rulison K. L. Loken E. (2009). I've fallen and I can't get up: Can high-ability students recover from early mistakes in CAT? Appl. Psychol. Measure. 33, 83–101. 10.1177/0146621608324023 20953275 PMC2954515

[B49] Spiegelhalter D. J. Thomas A. Best N. G. Lunn D. (2003). WinBUGS Version 1.4 User Manual. Cambridge: MRC Biostatistics Unit. Available online at: http://www.mrc-bsu.cam.ac.uk/wp-content/uploads/manual14.pdf

[B50] Spiegelhalter D. J. Best N. G. Carlin B. P. Van Der Linde A. (2002). Bayesian measures of model complexity and fit. J. R. Stat. Soc. Ser. B 64, 583–639. 10.1111/1467-9868.00353

[B51] Steinberg L. Thissen D. (1995). Item response theory in personality research, in Personality Research, Methods, and Theory: A Festschrift Honoring Donald W. Fiske, eds Shrout P. E. Fiske S. T. (Hillsdale, NJ: Erlbaum), 161–181.

[B52] Tanner M. A. Wong W. H. (1987). The calculation of posterior distributions by data augmentation. J. Am. Stat. Assoc. 82, 528–550. 10.1080/01621459.1987.10478458

[B53] Tavares H. R. de Andrade D. F. Pereira C. A. (2004). Detection of determinant genes and diagnostic via item response theory. Genet. Mol. Biol. 27, 679–685. 10.1590/S1415-47572004000400033

[B54] Tierney L. (1994). Markov chains for exploring posterior distributions (with discussions). Ann. Stat. 22, 1701–1762. 10.1214/aos/1176325750

[B55] Van der Linden W. J. Hambleton R. K. (eds.). (1997). Handbook of Modern Item Response Theory. New York, NY: Springer.

[B56] Waller N. G. Feuerstahler L. M. (2017). Bayesian modal estimation of the four-parameter item response model in real, realistic, and idealized data sets. Multivar. Behav. Res. 52, 350–370. 10.1080/00273171.2017.1292893 28306347

[B57] Waller N. G. Reise S. P. (2010). Measuring psychopathology with non-standard IRT models: fitting the four-parameter model to the MMPI, in Measuring Psychological Constructs: Advances in Modelbased Approaches, eds Embretson S. Roberts J. S. (Washington, DC: American Psychological Association), 147–173.

[B58] Yen Y.-C. Ho R.-G. Laio W.-W. Chen L.-J. Kuo C.-C. (2012). An empirical evaluation of the slip correction in the four parameter logistic models with computerized adaptive testing. Appl. Psychol. Measure. 36, 75–78. 10.1177/0146621611432862

[B59] Zhang Z. Hamagami F. Wang L. Grimm K. J. Nesselroade J. R. (2007). Bayesian analysis of longitudinal data using growth curve models. Int. J. Behav. Dev. 31, 374–383. 10.1177/0165025407077764

